# Reliability of Metformin’s protective effects against doxorubicin-induced cardiotoxicity: a meta-analysis of animal studies

**DOI:** 10.3389/fphar.2024.1435866

**Published:** 2024-08-08

**Authors:** Ming-Li Sun, Wei Chen, Xing-He Wang

**Affiliations:** ^1^ Phase I Clinical Trial Research Center, Beijing Shijitan Hospital Affiliated to Capital Medical University, Beijing, China; ^2^ Department of Intensive Care Unit, Beijing Shijitan Hospital Affiliated to Capital Medical University, Beijing, China

**Keywords:** animal models, Metformin, cardioprotective effect, DOX-induced cardiotoxicity, meta-analysis

## Abstract

**Background:**

The protective effects of metformin (Met) against doxorubicin (Dox)-induced cardiotoxicity via potential hypotheses of mechanisms of action with unknown reliability and credibility.

**Objectives:**

This study aimed to investigate the protective effects of Met against Dox-induced cardiotoxicity and the underlying mechanisms of action, as well as examine their reliability and credibility.

**Methods:**

A comprehensive search was conducted within the PubMed, Embase, Web of Science, Science Direct, Scopus, and CNKI databases from inception to 31 December 2023. Animal experiments evaluating the efficacy of Met against Dox-induced cardiotoxicity were included in this study. The primary efficacy outcomes were markers of myocardial injury. Effect size was measured using the standardized mean difference for continuous variables. Data were pooled using a random-effects model in the Stata 18 statistical software package.

**Results:**

Twenty-one studies involving 203–208 animals treated with Dox and 271–276 animals treated with Dox and Met were included in this analysis. Quality assessment revealed high-quality scores. Pooled results favored Met treatment based on the serum lactate dehydrogenase (LDH), creatine kinase-myocardial band (CK-MB), cardiac troponin I (cTnI), and aspartate aminotransferase levels. Sensitivity analysis using the leave-one-out method demonstrated stable results. Funnel plots, Egger’s test, and Begg’s test confirmed potential publication bias. The oxidative stress hypothesis has been investigated extensively based on abundant evidence.

**Conclusion:**

Met is effective and safe for protecting against Dox-induced cardiotoxicity, thus making it an appropriate drug for clinical investigation. The oxidative stress hypothesis of mechanism of action is well established with highest reliability and credibility.

## Introduction

Cancer is a globally recognized life-threatening and significant public health concern (Global Burden of Disease, 2019 Cancer Collaboration et al., 2022; Cao et al., 2021; Sung et al., 2021; Nemati et al., 2022). Doxorubicin (Dox) is a potent broad-spectrum chemotherapeutic agent possessing antitumor mechanisms that involve DNA helix insertion and the inhibition of topoisomerase II activity (Rivankar, 2014; Hulst et al., 2022). However, its clinical application is severely limited due to the occurrence of severe cardiotoxicity in approximately 11% of patients (Hanf et al., 2019; Chen et al., 2020; Xinyong et al., 2020; Arinno et al., 2021; Rawat et al., 2021; Dragojevic et al., 2022; Maleki et al., 2022; Alzokaky et al., 2023; Yi et al., 2023; Zhang et al., 2023).

Currently, dexrazoxane is the only medication approved by the Food and Drug Administration (FDA) for treating Dox-induced cardiotoxicity; however, it possesses notable disadvantages such as reduced efficacy of Dox and bone marrow suppression (Weiss et al., 1999; Mody et al. 2023; Spalato Ceruso et al., 2019; Ding et al., 2023). Therefore, there is an urgent need to identify novel drugs that can prevent and treat Dox-induced cardiotoxicity.

Metformin (Met) has been used for over 6 decades for the treatment of diabetes due to its multiple mechanisms of action and well-documented tolerability evidence (Flory and Lipska, 2019; Bertoluci et al., 2020; Shin et al., 2021; Bertoluci et al., 2023). Numerous studies have demonstrated the ability of Met to combat various cancers by inhibiting their growth, attenuating oxidative stress-induced cardiomyocyte apoptosis that plays a protective role against cardiac damage, reducing the incidence of cardiovascular events, and improving survival rates (Sasaki et al., 2009; Ferrannini and DeFronzo, 2015; Morales and Morris, 2015; Xie et al., 2020).

Several animal studies have demonstrated the protective effects of Met against Dox-induced cardiotoxicity via potential hypotheses of mechanisms of action involving oxidative stress reduction, energy preservation, apoptosis prevention, and autophagy regulation (Asensio-López et al., 2011; Ashour et al., 2012; Argun et al., 2016; Sheta et al., 2016; Zilinyi et al., 2018; Ajzashokouhi et al., 2020; Arinno et al., 2021; Singh et al., 2022; Zhang et al., 2023). The reliability and credibility of these mechanisms of action, however, remain unknown. Therefore, we conducted this systemic review and meta-analysis to investigate the protective effects of Met against Dox-induced cardiotoxicity and the underlying mechanisms of action, as well as examine their reliability and credibility.

## Materials and methods

This report adheres to the guidelines for Reporting of Systematic Reviews and Meta-analyses of Animal Experiments as well as the Preferred Reporting Items for Systematic Review and Meta-Analysis (PRISMA) guidelines (Peters et al., 2006; Moher et al., 2009; Percie du Sert et al., 2020; Page et al., 2021). The protocol is provided in Supplementary Material S1, Supplementary Appendix S1. This study was registered with the International Prospective Register of Systematic Reviews (PROSPERO) database under registration number CRD42022356210. Ethical approval was not required for this study because it solely involved the analysis of published documents.

### Literature search

The literature search focused on identifying published results from animal studies by searching databases that included PubMed, Embase, Web of Science, Science Direct, Scopus, and CNKI from inception to 31 December 2023, using the keywords “metformin” and “cardiotoxicity” (limited to “animal” without language restrictions) (Supplementary Material S1, Supplementary Appendix S2). Additional sources included grey (unpublished) literature obtained from computerized databases as well as published indexes/registries/meeting abstracts/conference proceedings, references/bibliographies, experts/research institutions/companies, or manufacturers related to the field being reviewed. Relevant reviews/editorials/reference lists were examined in additional relevant studies.

### Study selection

Studies meeting the following criteria were included: (1) utilization of animal models exhibiting Dox-induced cardiotoxicity; (2) treatment involving the administration of Met; and (3) controlled design regardless of randomization.

The exclusion criteria were as follows: (1) conference abstracts; (2) reviews; (3) studies not related to Dox-induced cardiotoxicity; (4) studies not related to the cardioprotective effects of Met; (5) studies not involving animal experiments; (6) corrigendum or editorial comments.

Two researchers independently screened the titles and abstracts to determine the relevance of the articles to the meta-analysis based on predefined inclusion and exclusion criteria. Prior to final selection, the full texts of potentially eligible studies were reviewed. Disagreements were resolved through consultation with a third researcher.

### Methodological quality and risk of bias (ROB) assessment

For all the included studies, quality and ROB analyses were conducted by two independent researchers. Disagreements were resolved through discussion with a third researcher until a consensus was reached.

Methodological quality was evaluated using the “Animal Research: Reporting of *in vivo* Experiment (ARRIVE) guidelines 2.0” that consist of the “ARRIVE Essential 10"(items 1–10) and the “Recommended Set” (items 11–21) (Percie du Sert et al., 2020). Each item is scored as 0 (not reported), 1 (reported but inadequate), or 2 (reported and adequate). A ratio between 0.75-1.00 indicated high quality, a ratio between 0.50-0.75 indicated intermediate quality, and a ration below 0.50 indicated low quality.

ROB was assessed using the Systematic Review Center for Laboratory Animal Experimentation (SYRCLE) tool that details six types of bias, including selection, performance, detection, attrition reporting, and other biases across ten domains (Hooijmans et al., 2014; Zeng et al., 2015). A judgment of “yes” indicated low ROB, and a judgement of “no” indicated high ROB. If insufficient details had been reported for proper assessment of ROB, then it would be judged as “unclear".

The kappa test was employed to assess interobserver consistency in both ARRIVE guidelines 2.0 and ROB evaluation. A high level of consistency was considered when the kappa value (*κ*) was ≥0.75, while a moderate level of consistency was indicated by *κ* values ranging from 0.40 to 0.75. A low level of consistency was observed when *κ* < 0.40.

### Data abstraction

Two researchers independently extracted data from the tables, graphs, or text. Discrepancies were resolved through consultation with a third researcher. If data were obtained from a graph, the average of the two values was calculated. In cases where the information in the included article was not clearly described, the authors were contacted via email, telephone, or other means to obtain missing or additional data.

### Study characteristics

The data extracted from the analyzed studies included animal model characteristics (species and weight), number of animals used in the experiments, experimental design details, treatment regimen information (method and dosage of drug administration), timing of drug administration, treatment outcomes/results, and other study attributes, such as author and publication year.

### Data synthesis

#### Parameters for evaluating the protective effects of Met against Dox-induced cardiotoxicity

To assess the protective effects of Met against Dox-induced cardiotoxicity, a meta-analysis was conducted on the following parameters: (1) body weight and the relative heart weight ratio; (2) myocardial injury markers, including serum/plasma lactate dehydrogenase (LDH), creatine kinase-myocardial band (CK-MB), cardiac troponin I (cTnI), and aspartate aminotransferase (AST); (3) cardiac function indicators such as brain natriuretic peptide (BNP) and N-terminal B-type natriuretic peptide (NT-proBNP) measured in laboratory tests and echocardiogram results, including left ventricular ejection fraction (LVEF) and left ventricular fractional shortening (LVFS), cardiac output (CO), and stroke volume (SV); (4) electrocardiogram findings such as QT interval, corrected QT interval (QTc interval), and heart rate.

### The mechanism underlying the protective effects exerted by Met against Dox-induced cardiotoxicity

The mechanisms underlying the protective effects of Met against Dox-induced cardiotoxicity were investigated using a comprehensive meta-analysis encompassing the following parameters:

(1) The oxidative stress hypothesis, including factors such as reactive oxygen species (ROS), superoxide dismutase (SOD) activity, cyclooxygenase-2 (COX-2), nitrites and nitrates (NO*x*), nitric oxide (NO), nitric oxide synthase (iNOS), DNA load of iNOS level, malondialdehyde (MDA), thiobarbituric acid-reactive substances (TBARS), glutathione (GSH), glutathione peroxidase (GPx), DNA load concentration of Matrix metalloproteinase 2 (MMP2), catalase, and catalase/β-actin mRNA level.
(2) The high-energy phosphate pool and alleviating energy starvation hypothesis involving adenosine triphosphate (ATP), blood glucose levels, mitochondrial swelling, CoA-SH, Acetyl-CoA, and adenosine 5′-monophosphate-activated protein kinase (AMPK).
(3) Apoptosis and autophagy hypothesis, including cardiomyocyte apoptosis rate, B-cell lymphoma-2 (Bcl-2), cardiac caspase-3, cleaved caspase-3, cleaved/pro-caspase-3, Beclin-1, LC3B, LC3B-II, LC3-II/LC3-I, mammalian target of rapamycin (mTOR), P62, Smad3, TGFβ1 DNA load level, and TGFβ1.
(4) The abnormal iron metabolism hypothesis, including the expression of ferritin heavy chain (FHC), total iron, and transferrin receptor (TfR) in cardiac tissue.

The primary efficacy outcomes included LDH, CK-MB, cTnI, and AST levels., which are commonly used markers of myocardial injury. Other parameters were considered as secondary outcomes in the meta-analysis. The effect size was measured using the standardized mean difference (SMD) for continuous variables. Due to the significant heterogeneity in the majority of the data, a random-effects model was employed for the meta-analysis (Halme et al., 2023).

### Investigation of heterogeneity in primary efficacy outcomes

Heterogeneity was assessed using the *I* ^2^ statistic and was categorized as no (<25%), low (25%–50%), moderate (50%–75%), or high (>75%) heterogeneity (Moher et al., 2009; Page et al., 2021). H- and Cochrane Q-tests were conducted to assess heterogeneity among the included studies. A value of H = 1 indicated no heterogeneity, H > 1.5 suggested identifiable heterogeneity, and H < 1.2 indicated homogeneity across all included studies. For the Q-test, a *p*-value <0.10 denoted significant heterogeneity among the included studies.

## Revealing the origins of heterogeneity in the primary efficacy outcomes

If significant heterogeneity (moderate or high, *I* ^2^ > 50%) was observed in the primary efficacy outcomes, sensitivity analyses were conducted to assess the robustness of the conclusions with respect to eligibility and analysis decisions. Sensitivity analysis was performed using the “leave-one-out” method (Zeng et al., 2015). Additionally, subgroup analysis and meta-regression were employed to explore any expected substantial heterogeneity. Subgroup analyses were performed based on potential sources of heterogeneity, including metformin dosage, type of cardiotoxicity (acute/chronic), animal species, and study designs.

### Assessment of reporting biases

The potential for publication bias was evaluated using funnel plots, Egger’s linear regression analysis, and Begg’s rank correlation test.

### Statistics and statistical software

The continuous measurement data are presented as the mean ± standard deviation. Statistical significance was defined as *p* < 0.05. The statistical software package Stata18 (Stata Corp., College Station, TX, USA) was used for meta-analysis.

## Results

### Description of studies

**FIGURE 1:**
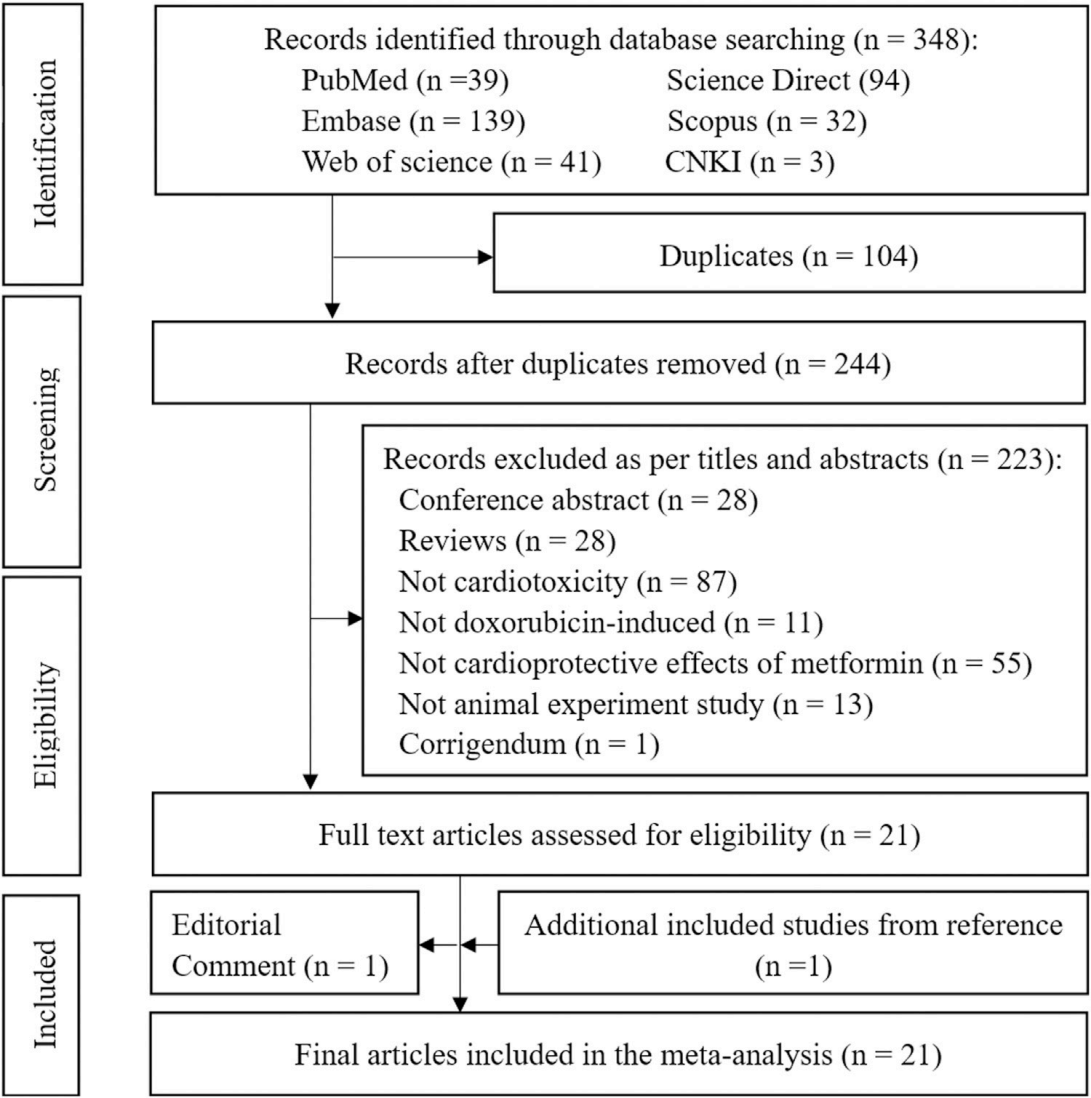
PRISMA flow diagram for identification of the eligible studies. Note: PRISMA = Preferred Reporting Items for Systematic Reviews and Meta-Analyses.

To relevance with treatment of Met in Dox-induced cardiotoxicity, we collected the 348 studies from the public databases such as PubMed (n = 39), Embase (n = 139), Web of Science (n = 41), ScienceDirect (n = 94), Scopus (n = 32), and CNK1 (n = 3). After removing duplicates and conducting title and abstract reviews, 21 full-text articles were assessed for eligibility. One article was excluded due to an editorial comment during the full-text evaluation, whereas one relevant study was included from the references of the included studies. Therefore, a final set of 21 articles were included in the meta-analysis (Figure 1).

The list and numbering of the included studies are both provided in Supplementary Material S1, Supplementary Appendix S3. The characteristics of these studies are summarized in Table 1 and Supplementary Material S1, Supplementary Appendix S4. A total of 203–208 animals, including Wistar rats (control, n = 89–92; treatment, n = 110–113 animals), Sprague–Dawley rats (control, n = 23–25; treatment, n = 35–37), mice (control, n = 61; treatment, n = 96), and rabbits (control, n = 30; treatment, n = 30).

**TABLE 1:** The characteristics of the included studies.

No.	Author (year)	Design	Animal species	Weight of animal	Number of animals	Experimental session
#1	Abdul Karim LZ (2021)	RCS	Healthy adult male Wistar rats	200–220 g	Acute Dox induction group: 6 Met + acute Dox induction group: 6	12 days
#2	Arinno A (2021)	RCS	Healthy male Wistar rats	about 350 g	Chronic Dox induction group: 8 Met + chronic Dox induction group: 8	30 days
#3	Ajmal K (2020)	RCS	Healthy adult male rabbits	average 2000 g	Acute Dox induction group: 6 Met + acute Dox induction group: 6	11 days
#4	Shaty MH (2019)	RCS	Healthy white male rabbits	600–2000 g	Acute Dox induction group: 6 Met + acute Dox induction group: 6 Chronic Dox induction group: 6 Met + chronic Dox induction group: 6	14 days
#5	Shaty MH (2018)	RCS	Healthy albino rabbits	600–2000 g	Acute Dox induction group: 6 Met + acute Dox induction group: 6 Chronic Dox induction group: 6 Met + chronic Dox induction group: 6	14 days
#6	Zilinyi R (2018)	RCS	Healthy adult female Sprague–Dawley rats	250–300 g	Chronic Dox induction group: 17–19 Met + chronic Dox induction group: 17–19	14 days
#7	Aruna P (2018)	CS	Healthy albino Wister rats	200–250 g	Acute Dox induction group: 6 Met + acute Dox induction group: 6 Met + acute Dox induction group: 6	15 days
#8	Argun M (2016)	RCS	Heathy male, 10-week-old Wistar albino rats	150–210 g	Chronic Dox induction group: 10 Met + chronic Dox induction group: 9	14 days
#9	Sheta A (2016)	RCS	Healthy adult male Wistar albino rats	160–200 g	Chronic Dox induction group: 8 Met + chronic Dox induction group: 8	7 days
#10	Shabrawy Abdo MEL (2016)	CS	Healthy adult male Wistar rats	200–250 g	Chronic Dox induction group: 24 Met + chronic Dox induction group: 24	21 days
#11	Kelleni MT (2015)	RCS	Healthy male Wistar rats	Not described	Chronic Dox induction group: 6–9 Met + chronic Dox induction group: 6–9	7 days
#12	Ashour AE (2012)	RCS	Healthy adult male Wistar albino rats	230–250 g	Chronic Dox induction group: 10 Met (50) + acute Dox induction group: 10 Met (500) + chronic Dox induction group: 10	11 days
#13	Mackay AD (2019)	RCS	Healthy 5-week-old male C57BL/6N mice	Mean: 18.7–19.1 g	Acute Dox induction group: 8 Met + acute Dox induction group: 7	9 days
#14	Ikewuchi JC (2021)	CS	Wistar rats	120–190 g	Acute Dox induction group: 5 Met + acute Dox induction group: 5	14 days
#15	Chen J (2020)	CS	Sprague–Dawley rats, males	200 ± 20 g	Chronic Dox induction group: 6 Met (25) + acute Dox induction group: 6 Met (100) +acute Dox induction group: 6 Met (400) +acute Dox induction group: 6	29 days
#16	Satyam SM (2023)	RCS	Healthy adult Wistar rats	150–200 g	Acute Dox induction group: 6 Met (180) + acute Dox induction group: 6 Met (250) + acute Dox induction group: 6	8 days
#17	Yi Y (2023)	RCS	Specific pathogen-free ICR mice (male), aged 5 weeks	22–25 g	Chronic Dox induction group: 8 Met (50) + chronic Dox induction group: 8 Met (100) + chronic Dox induction group: 8 Met (200) + chronic Dox induction group: 8 Chronic Dox induction group:5 Met (250) iv + chronic Dox induction group: 5 Met (500) iv + chronic Dox induction group: 5	21 days
#18	Alzokaky AA (2023)	RCS	Adult male albino mice	20–25 g	Acute Dox induction group: 5 Met (100) +acute Dox induction group: 5	14 days
#19	Wei J (2023)	RCS	6-week-old AMPKα2 knockout (AKO) mice	Not described	Acute Dox induction group (AKO): 6 Met (200) +acute Dox induction group (AKO): 6 Acute Dox induction group (WT): 6 Met (200) + acute Dox induction group (WT): 6	6 days
#20	Zhang S (2023)	RCS	6–8 weeks old male C57BL/6 mice	20–22 g	Chronic Dox induction group: 8 Met (100) + chronic Dox induction group: 8	28 days
#21	Kong L (2022)	RCS	Adult male C57BL/6 mice	25–30 g	Acute Dox induction group: 15 Met (50) + acute Dox induction group: 15 Met (100) + acute Dox induction group: 15	7 days

RCS, Randomized controlled study. i.p., intraperitoneal injection. Dox, Doxorubicin. Met, metformin.

## Methodological quality and ROB

In this study, 21 items were evaluated based on the ARRIVE guidelines 2.0. Of these, 16 studies (#2, 3, 6, 8–10, and 12-21) scored high in quality (76.19%, 16/21), whereas the remaining five studies (#1,4,5,7, and 11) were of intermediate quality (23.81%, 5/21). Items 1, 3, and 6–15 demonstrated high quality (57.14%,12/21), whereas items 2, 5, and 16-21 exhibited intermediate quality (38.10%,8/21). Item 4 was deemed low quality (4.76%,1/21). Overall, the included studies exhibited a high-level of quality with a ratio of quality score to maximum score equaling 0.79 (696/882). The assessment of ARRIVE guidelines by two observers revealed good agreement with *κ* = 0 .82 (*p* < 0 .001) (Supplementary Material S1, Supplementary Appendix S5).

Additionally, the ROB evaluation revealed that four studies (#3, 10, 13, and 16) possessed low ROB for “baseline characteristics” under the “selection bias” category; however, the rest of the domains and studies exhibited unclear ROB. In the category of “performance bias”, all studies with the exception of three with unclear risk of bias (#7, 10, and 14) in the domain of “random housing” exhibited low risk of bias, while all studies exhibited unclear risk of bias in the domain of “blinding”. Regarding “detection bias,” all studies possessed low ROB with the exception of three with unclear risk (#7, 10, and 14) in the domain of “random outcome assessment”, and only one study (#8) exhibited low risk while others were unclear in the domain of “blinding”. In terms of “attrition bias,” all studies indicated low risk with the exception of four with unclear risk (#2, 6, 13, and 19) in the domain of “incomplete outcome data”. For “reporting bias”, all studies demonstrated low risk with the exception of three with unclear risk (#2, 6, and 13) related to “selective outcome reporting”. In “other” categories, all studies exhibited low risk. Overall, there was a high-level of consistency between both observers when assessing the risks using the SYRCLE tool (*κ* = 0.87; *p* < 0.001) (Supplementary Material S1, Supplementary Appendix S6).

### Data synthesis of the primary efficacy outcomes and heterogeneity analysis

#### Lactate dehydrogenase

**FIGURE 2:**
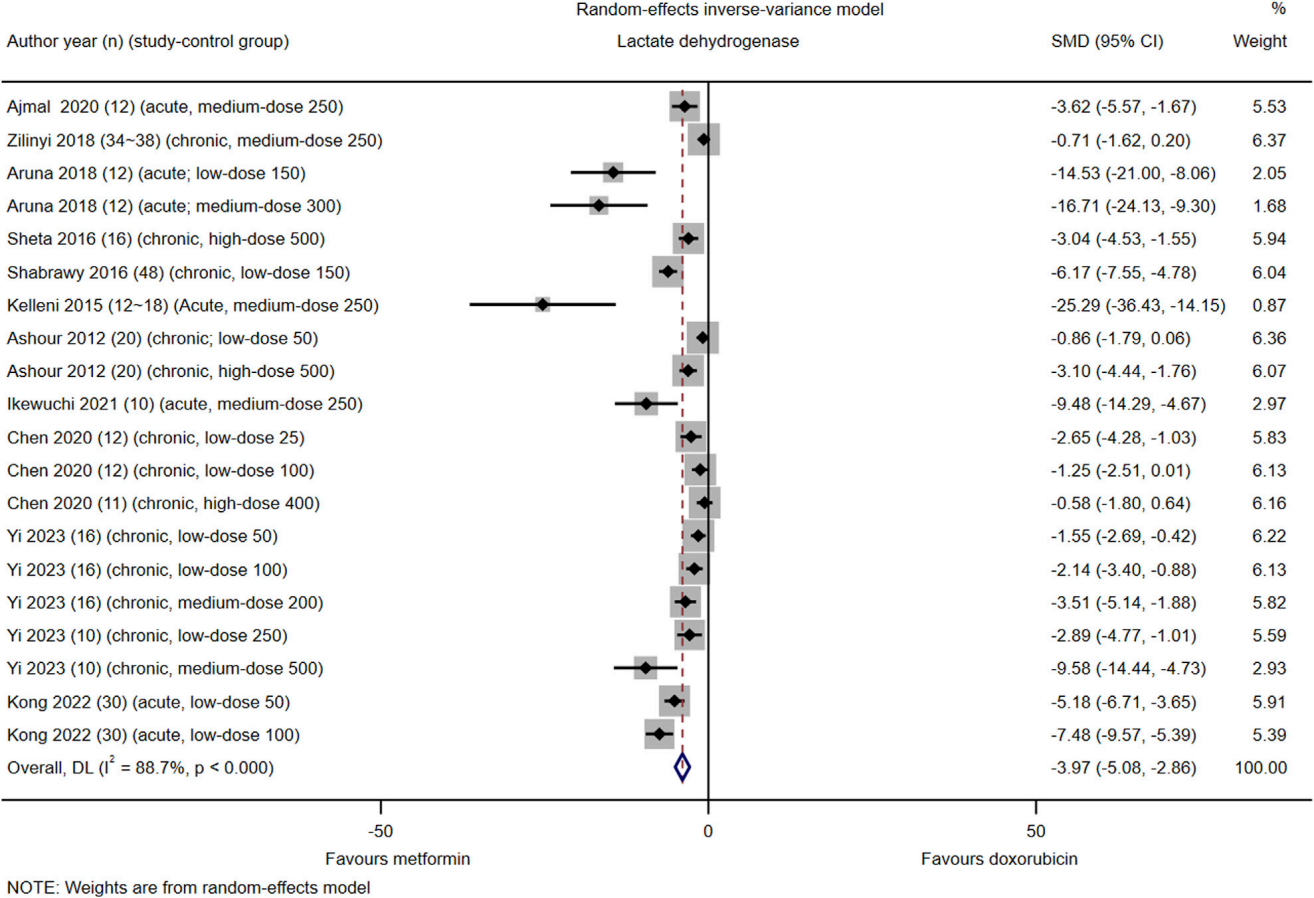
This Forest plot illustrates the comparison of serum lactate dehydrogenase (LDH) levels between metformin and placebo or black against Dox-induced cardiotoxicity.

Among the 21 included studies, 11 (#3, 6, 7, 9–12, 14, 15, 17, and 21) reported serum LDH levels in 20 control groups. The pooled effect size of serum LDH in favor of Met was −3.97 (95% CI = −5.08–−2.86; *Z* = −7.00, *p* < 0.001). Heterogeneity analysis revealed high heterogeneity for LDH (*I* ^2^ = 88.7%; *H* = 2.98; *Q* = 168.11, *p* < 0.001; *tau* ^2^ = 4.84) (Figure 2). Subgroup analysis did not identify the source of heterogeneity in LDH (Supplementary Material S1, Supplementary Appendix S7.1, Supplementary Figures S1–S4). Meta-regression analysis indicated that cardiotoxicity accounted for approximately 83.84% of the observed heterogeneity in LDH (*tau* ^2^ = 5.51, residual *I* ^2^ = 83.84%, adjusted *R* ^2^ = 58.04%, *p* = 0.004) (Supplementary Material S1, Supplementary Appendix S7.2).

### Creatine kinase-myocardial band

**FIGURE 3:**
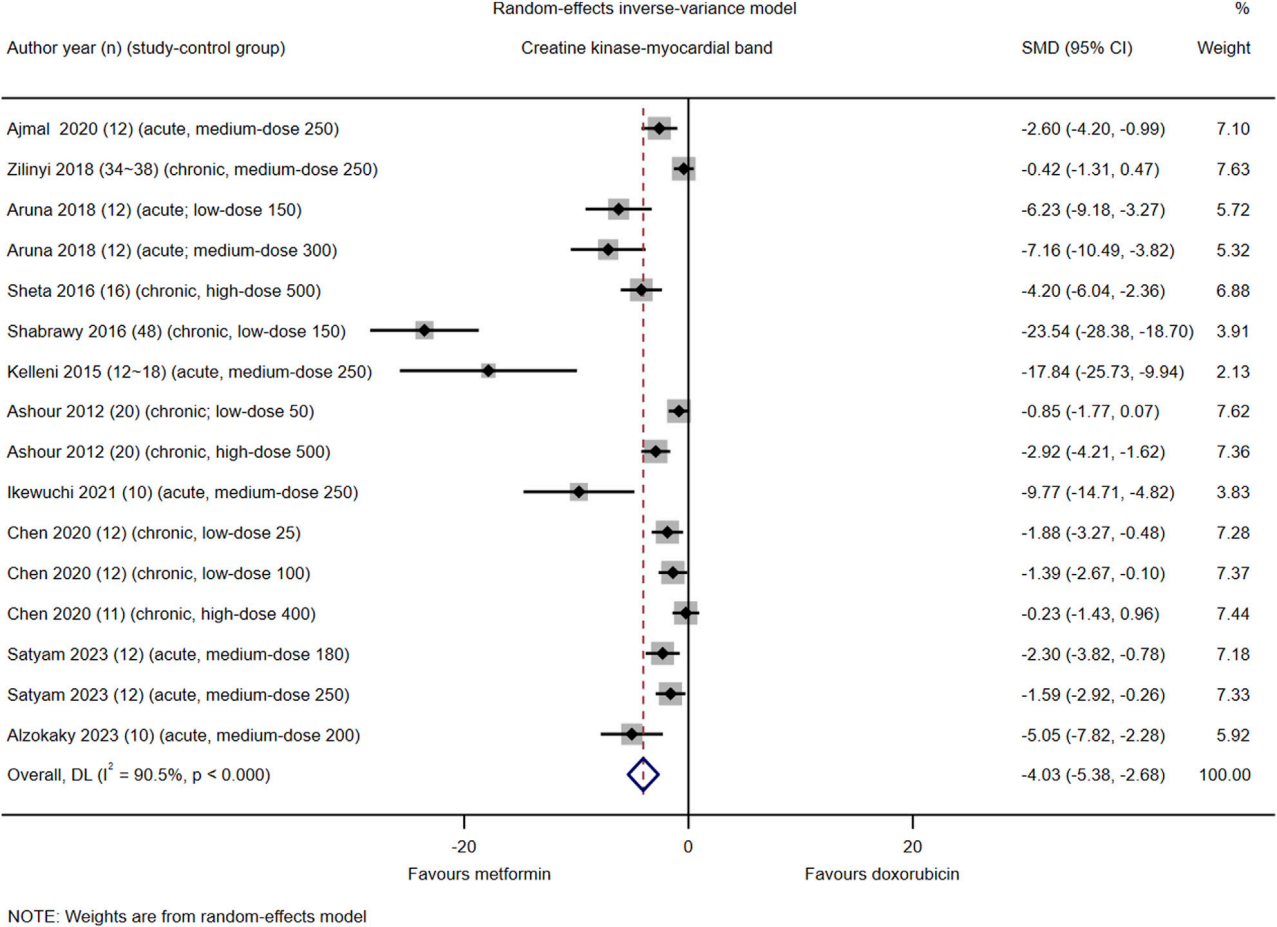
This Forest plot illustrates the comparison of serum creatine kinase-myocardial band (CK-MB) levels between metformin and placebo or black against Dox-induced cardiotoxicity.

Among the 21 included studies, serum CK-MB levels were reported in 16 control groups in 11 studies (#3, 6, 7, 9–12, 14-16, and 18). The pooled effect size of serum CK-MB in favor of Met was −4.03 (95% CI = −5.38–−2.68; *z* = −5.87, *p* < 0.001). Heterogeneity analysis revealed high heterogeneity for CK-MB (*I* ^2^ = 90.5%; *H* = 3.25; *Q* = 158.22, *p* < 0.001; *tau* ^2^ = 5.98) (Figure 3). Subgroup and meta-regression analyses did not identify a source of heterogeneity in CK-MB (Supplementary Material S1, Supplementary Appendix S8.1, Supplementary Figures S5–8, S8.2).

### Cardiac troponin I

**FIGURE 4:**
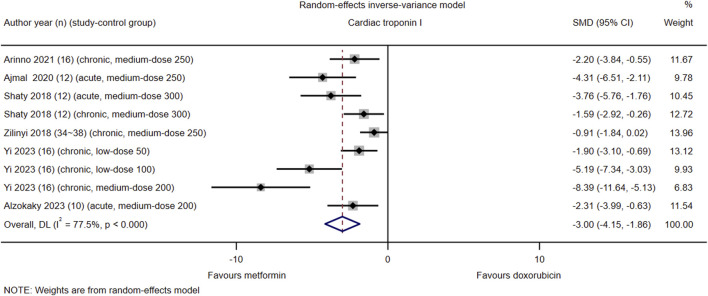
This Forest plot illustrates the comparison of serum cardiac troponin I (cTnI) levels between metformin and placebo or black against Dox-induced cardiotoxicity.

Among the 21 studies included, serum cTnI levels were reported in nine control groups of six studies (#2, 3, 5, 6, 17, and 18). The pooled effect size of serum cTnI in favor of Met was −3.00 (95% CI = −4.15–−1.86; *Z* = −5.15, *p* < 0.001). Heterogeneity analysis revealed high heterogeneity for cTnI (*I* ^2^ = 77.5%; *H* = 3.19; *Q* = 35.63, *p* < 0.001; *tau* ^2^ = 5.59) (Figure 4). Subgroup and meta-regression analyses did not identify the source of heterogeneity for cTnI (Supplementary Material S1, Supplementary Appendix S9, Supplementary Figures S9–S11).

### Aspartate aminotransferase

**FIGURE 5:**
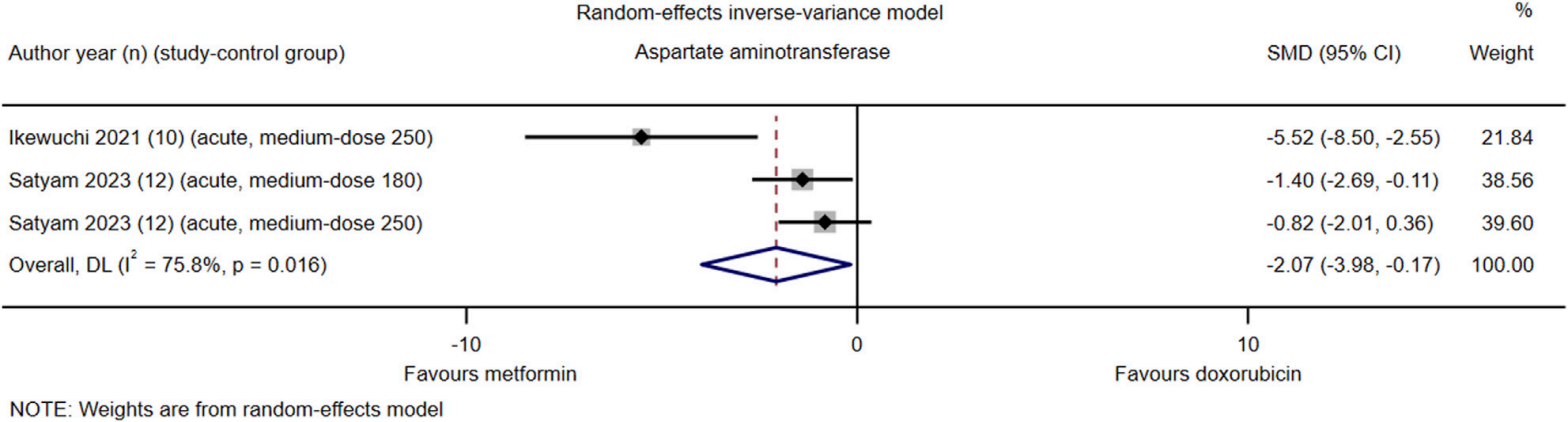
This Forest plot illustrates the comparison of serum aspartate aminotransferase (AST) levels between metformin and placebo or black against Dox-induced cardiotoxicity.

Among the 21 studies included, only two (#14 and #16) reported serum AST levels in their respective control groups. The pooled effect size of serum AST in favor of Met was −2.07 (95% CI = −3.98–−0.17; *z* = −2.13, *p* = 0.033). Heterogeneity analysis revealed high heterogeneity for AST (*I* ^2^ = 75.8%; *H* = 2.03; *Q* = 8.26, *p* = 0.016; *tau* ^2^ = 2.02) (Figure 5). Due to the limited number of included study-control groups, subgroup analysis and meta-regression analysis were not conducted.

### Sensitivity analysis for the primary efficacy outcomes

The stability of the pooled results for LDH, CK-MB, and cTnI was confirmed by sensitivity analysis using the leave-one-out method. (Supplementary Material S1, Supplementary Appendix S10, Supplementary Figures S12–S14).

### Data synthesis and heterogeneity analysis of secondary outcomes

#### Body weight and relative value of heart weight

Among the 21 included studies, seven (#10, 11, 13, 15, 18, 20, and 21) reported absolute body weight and/or relative heart weight values in a total of 14 study control groups. The pooled effect size for body weight favored Met with a value of 1.72 (95% CI = 0.36–3.07; *z* = 2.48, *p* = 0.013). Met treatment significantly improved the body weight of animals with Dox-induced cardiotoxicity; however, heterogeneity analysis revealed high heterogeneity in body weight (*I* ^2^ = 88.0%, *Q* = 50.03, *p* < 0.001). The pooled effect size for the ratio of heart-weight/tibial-length favored Met with a value of 2.71 (95% CI = 0.27–5.14, *z* = 2.18, *p* = 0.029) but exhibited high heterogeneity (*I* ^2^ = 90.4%, *Q* = 10.42, *p* = 0.001). When comparing the ratio of heart-weight/body-weight in animals with Dox-induced cardiotoxicity, Met-treated animals exhibited a small effect size (SMD = −1.14, 95%CI = −1.95–−0.33, *z* = −2.77, *p* = 0.006) with no heterogeneity (*I* ^2^ = 0.0%, *Q* = 0.00, *p* = 0.983). Based on data from the three control groups reporting heart/brain weight ratios, the pooled effect size indicated no significant benefits from Met (SMD = 0.52, 95% CI = −0.22 – 1.25, *z* = 1.38, *p* = 0.167) and no heterogeneity (*I* ^2^ = 11.0%, *Q* = 2.25, *p* = 0.325). (Supplementary Material S1, Supplementary Appendix S11, Supplementary Figure S15).

These findings suggest that Met improves heart weight significantly more than it does tibial length but less than overall body weight, and it exerts an equivalent impact on brain weight.

### Cardiac function

Among the 21 included studies, eight (#2, 6, 8, 15, 17, and 19-21) assessed cardiac function levels using echocardiography. The pooled effect sizes for left ventricular ejection fractions, fractional shortening, cardiac output, and stroke volume in favor of Met were as follows: 2.49 (95%CI = 1.54–3.44; *z* = 5.14, *p* < 0.001) exhibiting high heterogeneity (*I* ^2^ = 82.0%*Q* = 55.55, *p* < 0.001); no heterogeneity observed for a value of 1.72 (95% CI = 1.24–2.21; *z* = 6.95, *p* < 0.001; *I* ^2^ = 10.4%, *Q* = 8.93, *p* = 0.349); a value of 3.12 (95%CI = 1.48–4.77; *z* = 3.72, *p* < 0.001; *I* ^2^ = 86.1%, *Q* = 28.72, *p* < 0.001); a value of 0.74 (95%CI = 0.13–1.56; *z* = 2.37, *p* = 0.018; *I* ^2^ = 0.0%, *Q* = 0.43, *p* = 0.511), respectively (Supplementary Material S1, Supplementary Figure S16).

Although laboratory indicators of cardiac function such as BNP, NT-proBNP, and atrial natriuretic peptide were reported in two studies (#2 and #8), only one study with control group data was available for each indicator. Therefore, a meta-analysis could not be conducted for these indicators.

### Characteristics of electrocardiograms and heart rates

Among the 21 included studies, five studies (#2, 6, 10, 15, and 16) provided data regarding electrocardiogram characteristics and heart rates. The meta-analysis revealed significant pooled effect sizes favoring Met for QT interval (−2.04; 95% CI = −3.89–−0.19; *z* = −2.16, *p* = 0.031) with high heterogeneity (*I* ^2^ = 77.2%, *Q* = 8.77, *p* = 0.012), QTc interval (−1.85; 95% CI = −2.83–−0.88; *z* = −3.74, *p* < 0.001) with moderate heterogeneity (*I* ^2^ = 54.7%, *Q* = 8.84, *p* = 0.065), and heart rate improvement (1.54; 95%CI = 0.38–−2.69; *z* = 2.60, *p* = 0.009) with high heterogeneity (*I* ^2^ = 77.5%; H = 2.11; *Q* = 22.25, *p* < 0.001; *tau* ^2^ = 1.44). Animals treated with Met exhibited a significant reduction in QT and QTc intervals as well as an improvement in heart rate compared to that in animals with Dox-induced cardiotoxicity (Supplementary Material S11, Supplementary Figure S17).

### Oxidative stress hypothesis

Of the 21 included studies, 14 (#1, 2, 4, 6, 8-15 and 18, 20) reported biomarkers for oxidative stress. The pooled effect size for malondialdehyde in cardiac tissue was −4.05 (95%CI = −6.21–−1.89, *z* = −3.67, *p* < 0.001); however, there was high heterogeneity (*I* ^2^ = 82.3%, *Q* = 28.28, *p* < 0.001). For malondialdehyde in serum alone, a significant effect size of −4.43 (95%CI = −5.50–−3.37, *z* = −8.15, *p* < 0.001) was observed without heterogeneity (*I* ^2^ = 16.0%, *Q* = 5.95, *p* = 0.311). However, for thiobarbituric acid reactive substances in the heart, a moderate effect size of −1.20 (95%CI = −2.73–−0.34, *z* = −1.53, *p* = 0.127) was observed with moderate heterogeneity (*I* ^2^ = 67.5%, *Q* = 3.08, *p* = 0.079). (Supplementary Material S11, Supplementary Figure S18A).

The pooled effect size of TNFα favored Met with a value of-2.32 (95% CI = −3.70–−0.94; *z* = −3.29, *p* = 0.001) and exhibited high heterogeneity (*I* ^2^ = 76.7%, *Q* = 17.14, *p* = 0.002). For the DNA load of iNOS level, the pooled effect size in favor of Met was observed to be −5.80 (95% CI = −7.79–−3.82; *z* = −5.73, *p* < 0.001) without any observed heterogeneity (*I* ^2^ = 0.0%, *Q* = 0.71, *p* = 0.401). However, for the DNA load concentration of MMP2 level, the pooled effect size was determined as −16.19 (95% CI = −41.28 – 8.91; *z* = −1.26, *p* = 0.206), and this was accompanied by high heterogeneity (*I* ^2^ = 93.0%, *Q* = 14.27, *p* < 0.001). (Supplementary Material S11, Supplementary Figure S18B).

The pooled effect size of serum reduced glutathione in favor of Met was 1.89 (95%CI = 0.93–2.86, *z* = 3.84, *p* < 0.001) with moderate heterogeneity (*I* ^2^ = 50.5%, *Q* = 8.08, *p* = 0.089), thus indicating a statistically significant difference. For cardiac reduced glutathione, the pooled effect size favoring Met was determined to be 3.49 (95%CI = 1.70–5.28, *z* = 3.83, *p* < 0.001) with high heterogeneity (*I* ^2^ = 82.0%, *Q* = 22.27, *p* < 0.001), thus suggesting substantial variability among studies. For superoxide dismutase, the effect size was estimated at 2.64 (95%CI = 1.31–3.98, *z* = 3.89, *p* < 0.001), also indicating high heterogeneity (*I* ^2^ = 80.5%, *Q* = 35.98, *p* < 0.001). No significant association was observed between Met and catalase levels after treatment compared to values without treatment (effect size: 5.96 [95% CI = −7.11 – 19.02; *z* = 0.89, *p* = 0.372]), but there was high heterogeneity among studies (*I* ^2^ = 93.7%, *Q* = 15.87, *p* < 0.001). Similarly, no significant difference in catalase/β-actin mRNA expression between groups (effect size: 1.77 [95% CI = −1.51 – 5.04; *z* = 1.06, *p* = 0.290]) was detected, and again there was high heterogeneity among studies (*I* ^2^ = 93.4%, *Q* = 15.12, *p* < 0.001). Finally, the analysis indicated no significant variation in glutathione peroxidase levels between groups (effect size: 5.78 [95% CI = −8.03 – 19.58; *z* = 0.82, *p* = 0.412]), and there was high heterogeneity among studies (*I* ^2^ = 94.2%, *Q* = 17.21, *p* < 0.001) (Supplementary Material S11, Supplementary Figure S18C).

Certain oxidative stress biomarkers such as nitric oxide, nitrites, nitrates, inducible nitric oxide synthase, cyclooxygenase-2, ferritin heavy chain, total iron content in the heart, and transferrin receptors could not be included in the meta-analysis.

### Alleviating energy starvation and preserving mitochondrial function hypothesis

Among the 21 studies included, nine (#1, 2, 6, 9, 10, 12, 16, 18, and 20) reported energy metabolism and mitochondrial function. The pooled effect sizes for ATP, intramitochondrial CoA-SH, and Phospho-AMPK/AMPK in favor of Met were estimated as follows: ATP pooled effect size = 2.85 (95%CI = −0.08 – 5.78, *z* = 1.91, *p* = 0.057) with high heterogeneity (*I* ^2^ = 90.7%, *Q* = 21.44, *p* < 0.001); intramitochondrial CoA-SH pooled effect size = 1.54 (95%CI = 0.83–2.26, *z* = 4.22, *p* < 0.001) with no heterogeneity (*I* ^2^ = 0.0%, *Q* = 0.27, *p* = 0.604); Phospho-AMPK/AMPK pooled effect size = 2.65 (95%CI = −1.71 – 7.00, *z* = 1.19, *p* = 0.233) with high heterogeneity (*I* ^2^ = 90.5%, *Q* = 10.47, *p* = 0.001) (Supplementary Material S11, Supplementary Figure S19A).

The pooled effect sizes of blood glucose levels and intramitochondrial Acetyl-CoA in favor of Met were estimated as follows: blood glucose levels-pooled effect size = −1.23 (95%CI = −2.74 – 0.27, *z* = −1.61, *p* = 0.107) with high heterogeneity (*I* ^2^ = 88.9%, *Q* = 45.06, *p* < 0.001); intramitochondrial Acetyl-CoA-pooled effect size = −1.77 (95%CI = −3.28–−0.26, *z* = −2.30, *p* = 0.021) with moderate heterogeneity (*I* ^2^ = 74.1%, *Q* = 3.85, *p* = 0.050) (Supplementary Material S11, Supplementary Figure S19B).

Additional markers of energy metabolism and mitochondrial function such as mitochondrial swelling, mitochondrial membrane potential, respiratory control ratio, and intramitochondrial ROS were not included in the meta-analysis due to limited availability of study-control group data for each indicator.

### Apoptosis hypothesis

Among the 21 studies included, eight (#2, 5, 9, 11, 15, and 18, 20, 21) reported apoptosis markers. The pooled effect sizes favoring Met were as follows: Bax/Bcl-2 (−1.62; 95%CI = −2.68–−0.57, *z* = −3.02, *p* = 0.003) with moderate heterogeneity (*I* ^2^ = 61.0%, *Q* = 10.26, *p* = 0.036); cardiac caspase-3 (−4.78; 95%CI = −6.84–−2.72, *z* = −4.55, *p* < 0.001) with high heterogeneity (*I* ^2^ = 83.6%, *Q* = 24.33, *p* < 0.001); cleaved caspase-3 (−5.44; 95%CI = −7.93–−2.95, *z* = −4.28, *p* < 0.001) with moderate heterogeneity (*I* ^2^ = 80.7%, *Q* = 10.35, *p* = 0.006); cleaved/Pro-caspase-3 (-2.91; 95%CI = −3.84–−1.98, *z* = −6.16, *p* < 0.001) with moderate heterogeneity (*I* ^2^ = 21.0%, *Q* = 5.06, *p* = 0.281); DNA load of TGFβ1 level (−11.05; 95%CI = −19.50–−2.60, *z* = −2.56, *p* = 0.010) with moderate heterogeneity (*I* ^2^ = 78.8%, *Q* = 4.71, *p* = 0.030); Serum SMAD3 (−7.42; 95%CI = −19.63–−4.79, *z* = −1.19, *p* = 0.234) with moderate heterogeneity (*I* ^2^ = 93.2%, *Q* = 14.62, *p* < 0.001); apoptotic index/rate (−5.87; 95%CI = −8.14–−3.59, *z* = −5.06, *p* < 0.001) with moderate heterogeneity (*I* ^2^ = 70.8%, *Q* = 6.85, *p* = 0.033) (Supplementary Material S11, Supplementary Figure S20).

### Autophagy hypothesis

Among the 21 included studies, three (#2, # 6, and # 20) reported the presence of autophagic markers. The pooled effect sizes of Beclin-1/GAPDH, P62/GAPDH, and LC3-II/LC3-I in favor of Met were −0.31 (95% CI = −3.10 – 2.47; *z* = −0.22, *p* = 0.825) with high heterogeneity (*I* ^2^ = 85.7%, *Q* = 6.98, *p* = 0.008), −3.83 (95% CI = −7.92 – 0.26; *z* = −1.84, *p* = 0.066) with high heterogeneity (*I* ^2^ = 82.9%, *Q* = 5.86, *p* = 0.015), and 2.88 (95% CI = −1.35 – 7.11; *z* = 1.34, *p* = 0.182) with high heterogeneity (*I* ^2^ = 92.4%, *Q* = 26.17, *p* < 0.001) (Supplementary Material S11, Supplementary Figure S21).

However, due to the limited number of study controls (n = 2), although Met demonstrated an increasing trend for Beclin-1/GAPDH, P62/GAPDH, and LC3-II/LC3-I, no statistically significant differences were observed compared to these values associated with Dox treatment. Furthermore, certain autophagic markers such as beclin-1, LC3B, p62, and p-TOR/mTOR could not be meta-analyzed due to insufficient data from only one control group for each indicator.

### Publication bias

Funnel plots of serum LDH, CK-MB, and cTnI levels exhibited clear asymmetry, thus indicating the presence of significant publication bias (Supplementary Material S1, Supplementary Appendix S12, Supplementary Figures S22–S24). Publication bias was confirmed using Egger linear regression (*p* < 0.001) and the Begg rank correlation test (Kendall score, z, and *p*-value with continuity correction = −126, 4.06, and <0.001 for LDH; −104, 4.64, and <0.001 for CK-MB; −32, 3.23, and 0.001 for cTnI, respectively). (Table 2).

**TABLE 2:** The results of Egger linear regression and Begg rank correlation test.

Item	Egger linear regression				Begg rank correlation				
	Coef	95% CI	t	*p*	adj. Kendall score (P-Q)		SD	*z* ^a^	*p* ^a^
LDH	−5.22	−7.12–−3.12	−5.76	<0.001	−126		30.82	4.06	<0.001
CK-MB	−6.22	−8.00–−4.44	−7.48	<0.001	−104		22.21	4.64	<0.001
CTnI	−5.72	−7.17–−4.26	−9.30	<0.001	−32		9.59	3.23	0.001

LDH, serum lactate dehydrogenase; CK-MB, serum creatine kinase-myocardial band. cTnI, cardiac troponin I, CI, confidence interval; SD, standard deviation of score.

^a^ continuity corrected.

## Discussion

### The main findings and scientific significance

To the best of our knowledge, this is the first comprehensive evaluation of the protective effects of Met against Dox-induced cardiotoxicity and its underlying mechanisms in animal experiments. Our systematic review and meta-analysis provide compelling evidence supporting the cardioprotective effects of Met in animal models of Dox-induced cardiotoxicity, thus confirming its mode of action.

Additionally, we conducted a comprehensive review of existing hypotheses concerning the mechanisms underlying the cardioprotective effects of Met against Dox-induced cardiotoxicity. Our analysis revealed that the oxidative stress hypothesis has been extensively investigated and supported by abundant evidence, and his reliability and credibility is the highest. The alleviation of energy starvation and improvement in the mitochondrial function hypothesis emerged as the second most explored, while the apoptosis hypothesis ranked third in terms of research attention. However, the current evidence is inadequate to substantiate this hypothesis, and additional research is warranted. It is worth noting that due to intricate interplay between various pathways, they are intricately interconnected with each other. For example, AMPK not only participates in energy metabolism and preservation of mitochondrial function but also plays a pivotal role in regulating oxidative stress, apoptosis, and autophagy (Ashour et al., 2012; Mackay et al., 2015; Chen et al., 2020; Alzokaky et al., 2023; Zhang et al., 2023). Once activated, AMPK primarily regulates four major types of metabolism in mammals, including protein metabolism, lipid metabolism, carbohydrate metabolism, autophagy, and mitochondrial homeostasis, encompassing almost all physiological metabolic activities of living organisms (Steinberg and Hardie, 2023).

Met administration effectively inhibited oxidative stress, improved mitochondrial function, and enhanced energy supply while mitigating apoptosis and autophagy. Consequently, it exerted a protective effect against Dox-induced cardiotoxicity in various animal models. These findings highlight the potential utility of Met as an adjunct therapy to alleviate the cardiac side effects associated with Dox treatment in cancer patients.

Despite the significant heterogeneity among the included studies, sensitivity analysis demonstrated robustness in the pooled results for primary efficacy outcomes. Furthermore, meta-regression analysis revealed that the type of cardiotoxicity was a source of heterogeneity in LDH levels. However, all animal models within each subgroup benefited from Met treatment, ultimately resulting in reduced Dox-induced cardiotoxicity. Subgroup analysis and meta-regression indicated no heterogeneity among the primary efficacy outcomes attributed to the animal species involved. This suggests that the protective effect of Met against Dox-induced cardiotoxicity may occur in multiple species.

### Interpretation of the results considering the totality of available evidence

Dox-induced cardiotoxicity occurs via multiple mechanisms, including enhanced production of ROS, peroxidation of cardiolipin, mitochondrial injury, impaired mitochondrial biogenesis, accumulation of iron, DNA damage, and dysfunction of autophagy/mitophagy. These processes ultimately result in either inadequate or excessive elimination of damaged mitochondria, thus exacerbating the cardiac injury (Rawat et al., 2021).

Recently, numerous strategies have been developed to prevent and treat Dox-induced cardiotoxicity (Rawat et al., 2021). However, Met provided the best evidence for protection against Dox-induced cardiotoxicity due to its comprehensive mechanism (Ashour et al., 2012; Ajzashokouhi et al., 2020; Arinno et al., 2021; Singh et al., 2022). Met protects against Dox-induced cardiotoxicity by attenuating ROS generation and oxidative stress, inhibiting mitochondrial damage, preserving energy production, and reducing apoptosis and autophagy. Furthermore, Met not only mitigates Dox-induced cardiotoxicity but also enhances its antitumor effects by suppressing cancer stem cell activity, downregulating the expression of the drug-resistant gene P-glycoprotein, and inducing apoptosis via inhibition of the mammalian target of rapamycin signaling pathway while activating AMPK (Singh et al., 2022).

## Strengths

First, our findings validate the protective effects of Met against Dox-induced cardiotoxicity and present a comprehensive systematic review and meta-analysis to elucidate the underlying mechanisms responsible for these protective effects, and exam the reliability and credibility of various hypotheses of the existing mechanism of action. Furthermore, Met exhibits anticancer properties and enhances the antitumor effects of doxorubicin (Morales and Morris, 2015; Xie et al., 2020; Singh et al., 2022). Third, Met is distinguished by its exceptional safety profile, high efficacy, and affordability (Sun et al., 2022). Consequently, it is anticipated to play an increasingly significant role in clinical practice in the foreseeable future.

## Implications of these findings in practice

The robust findings from this study enhance the level of evidence in animal models and may facilitate the translation from preclinical experiments to subsequent clinical trials. Wei reported that chemotherapy with Dox causes cardiotoxicity, which can be mitigated by combined treatment with Met possibly through a mechanism involving the AMPK pathway (Wei et al., 2023). However, others hold a different perspective. Osataphan reported that in breast cancer patients treated with doxorubicin, co-treatment with Met did not prevent myocardial injury (Osataphan et al., 2023). Interestingly, despite Osataphan’s negative findings, he observed that Dox impaired mitochondrial function at the cellular level, and Met was the sole intervention capable of preserving mitochondrial respiration during Dox therapy (Osataphan et al., 2023). Osataphan also suggested that future studies should explore potential cardioprotective effects of Met (Osataphan et al., 2023). Although there is limited clinical evidence regarding the cardioprotective effects of Met against Dox-induced cardiotoxicity, we have reasons to anticipate that due to its inherent advantages in terms of safety, efficacy, and cost-effectiveness, Met is poised to surpass dexrazoxane as a more safe and effective medication for Dox-induced cardiotoxicity.

## Limitations

The present meta-analysis has a few limitations. First, potential publication bias was confirmed using funnel plots and Egger’s and Begg’s tests. Second, a significant heterogeneity was observed among the included studies. Third, this study was based on animal experiments, and this may have overlooked important considerations such as randomization, masked treatment allocation, and blinded outcome assessment. Additionally, this analysis relies solely on data from animal experiments and lacks clinical relevance. Therefore, additional clinical research is warranted to validate these findings, optimize metformin administration schedules and dosages, and evaluate its long-term effectiveness and safety. Last but not least, the number of animals included seems relatively small for a meta-analysis, which may limit the statistical power of the analysis.

## Conclusion

The present study systematically reviewed available peer-reviewed literature detailing the efficacy and safety of Met in the context of mitigating Dox-induced cardiotoxicity. Based on compelling evidence from animal experiments, Met has demonstrated both effectiveness and safety in protecting against Dox-induced cardiotoxicity, thus representing a promising therapeutic and preventive option for individuals at risk of developing this condition. Extensive studies have been conducted examining the oxidative stress hypothesis with highest reliability and credibility, and have yielded abundant evidence supporting its role in Dox-induced cardiotoxicity. Moreover, the well-established cardioprotective mechanisms of Met strengthen its potential as a viable interventional strategy. However, it is imperative to validate these findings using rigorous clinical randomized controlled trials.

## Data Availability

The original contributions presented in the study are included in the article/Supplementary Material, further inquiries can be directed to the corresponding authors.
