# Supplementary material for: Reliability of Metformin’s protective effects against doxorubicin-induced cardiotoxicity: a meta-analysis of animal studies
Source: Front Pharmacol. 2024 Aug 8;15:1435866. doi: 10.3389/fphar.2024.1435866 (PMC11338926; doi:10.3389/fphar.2024.1435866)
Supplement: Supplementary file 1 [file DataSheet1.pdf]

Reliability of Metformin's Protective Effects against Doxorubicin-induced Cardiotoxicity: A meta-analysis of Animal Studies

Supplementary 1

Appendix

Content

1. The protocol of this study..1

2. Literature retrieval 4

2.1 Embase database searching 4

2.2 PubMed literature searching..4

2.3 Web of Science searching 5

2.4 Science Direct searching 6

2.5 Scopus searching 6

2.6 CNKI searching 7

3. Literature included in this study 8

4. The characteristics of the included studies (continue) 11

5. Quality assessment according to ARRIVE guidelines 2.0 18

6. Risk of Bias (ROB) assessing with SYRCLE's RoB tool 20

7. Subgroup- and meta-regression analysis for serum lactate dehydrogenase (LDH) 21

7.1 Subgroup analysis for serum LDH 21

Fig. S1 Subgroup analysis according to dose of metformin for serum LDH 21

Fig. S2 Subgroup analysis according to type of cardiotoxicity for serum LDH 22

Fig. S3 Subgroup analysis according to species of animal for serum LDH 23

Fig. S4 Subgroup analysis according to study designs for serum LDH 24

7.2 Meta-regression analysis for serum LDH 25

8. Subgroup- and meta-regression analysis for serum creatine kinase-myocardial band (CK-MB)..26

8.1 Subgroup analysis for serum creatine CK-MB 26

Fig. S5 Subgroup analysis according to dose of metformin for serum CK-MB 26

Fig. S6 Subgroup analysis according to type of cardiotoxicity for serum CK-MB 27

Fig. S7 Subgroup analysis according to species of animal for serum CK-MB 28

Fig. S8 Subgroup analysis according to study designs for serum CK-MB 29
8.2 Meta-regression analysis for serum creatine CK-MB ……………………................…30
9. Subgroup- and meta-regression analysis for serum cardiac troponin I (cTnI) ……………........…31
9.1 Subgroup analysis for serum cTnI ………………….…………………………..........…31
Fig. S9 Subgroup analysis according to dose of metformin for serum cTnI ....….31
Fig. S10 Subgroup analysis according to type of cardiotoxicity for serum cTnI ....….32
Fig. S11 Subgroup analysis according to species of animal for serum cTnI 33
9.2 Meta-regression analysis for serum cTnI ……………………………….................…34
10. Sensitivity analysis of primary efficacy outcomes 35
Fig. S12 Sensitivity analysis using "leave-one-out" method for serum LDH 35
Fig. S13 Sensitivity analysis using "leave-one-out" method for serum CK-MB 36
Fig. S14 Sensitivity analysis using "leave-one-out" method for serum cTnI 37
11. Data synthesis and heterogeneity analysis of secondary outcomes 38
Fig. S15 Forest plot of body weight and relative value of heart weight 38
Fig. S16 Forest plot of cardiac function 39
Fig. S17 Forest plot of characteristics of electrocardiogram and heart rates 40
Fig. S18 Forest plot of oxidative stress hypothesis ………...41
Fig. S19 Forest plot of alleviating energy starvation and preserving mitochondrial function hypothesis ……………………………………………………………………………....43
Fig. S20 Forest plot of apoptosis hypothesis ………………………………………….......44
Fig. S21 Forest plot of autophagy hypothesis …...…………………………….................…45
12. Funnel plot for publication bias of the primary efficacy outcomes...............................….......46
Fig. S22 serum lactate dehydrogenase (LDH) ....….......46
Fig. S23 serum creatine kinase-myocardial band (CK-MB) 47
Fig. S24 serum cardiac troponin I (cTnI) ....…..48

1. Protocol

1. Background: Cancer is a globally recognized life-threatening and significant public health concern. Doxorubicin (Dox) is a potent broad-spectrum chemotherapeutic agent. However, its clinical application is severely limited due to the occurrence of severe cardiotoxicity in approximately 11% of patients. Currently, dexrazoxane is the only medication approved by the Food and Drug Administration for treating DOX-induced cardiotoxicity; however, it possesses notable disadvantages such as reduced efficacy of Dox and bone marrow suppression. Therefore, there is an urgent need to identify novel drugs that can prevent and treat DOX-induced cardiotoxicity. Several animal studies have demonstrated the protective effects of metformin (Met) against DOX-induced cardiotoxicity. However, variations exist among these studies in terms of the animal models used, the drug doses administered, the types of observed cardiotoxicities examined, and the different indicators measured. In light of this, conducting a systematic review and meta-analysis investigating the efficacy of Met against Dox-induced cardiotoxicity becomes imperative.

2. Objectives: This study aimed to investigate the protective effects of Met against DOX-induced cardiotoxicity and the underlying mechanisms through a systematic review and meta-analysis of existing animal studies.

3. Methods:

3.1 Literature search: The literature search focused on identifying published results from animal studies by searching databases that included PubMed, Embase, Web of Science, Science Direct, Scopus, and CNKI from inception to December 31st, 2023, using the keywords "metformin" and "cardiotoxicity" (limited to "animal" without language restrictions). Additional sources included grey (unpublished) literature obtained from computerized databases as well as published indexes/registries/meeting abstracts/conference proceedings, references/bibliographies, experts/research institutions/companies, or manufacturers related to the field being reviewed. Relevant reviews/editorials/reference lists were examined in additional relevant studies.

3.2 Study selection

3.2.1 Inclusion Criteria: (1) utilization of animal models exhibiting DOX-induced cardiotoxicity; (2) treatment involving the administration of Met; and (3) controlled design regardless of randomization.

3.2.2 Exclusion criteria: (1) conference abstracts; (2) reviews; (3) studies not related to DOX-induced cardiotoxicity; (4) studies not related to the cardioprotective effects of Met; (5) studies not involving animal experiments; (6) corrigendum or editorial comments.

Two researchers independently screened the titles and abstracts to determine the relevance of the articles to the meta-analysis based on predefined inclusion and exclusion criteria. Prior to final selection, the full texts of potentially eligible studies were reviewed. Disagreements were resolved through consultation with a third researcher.

3.3 Methodological quality and risk of bias (ROB) assessment: For all the included studies, quality and ROB analyses were conducted by two independent researchers. Disagreements were

resolved through discussion with a third researcher until a consensus was reached.

Methodological quality was evaluated using the "Animal Research: Reporting of in vivo Experiment (ARRIVE) guidelines 2.0" that consist of the "ARRIVE Essential 10" (items 1–10) and the "Recommended Set" (items 11–21). Each item is scored as 0 (not reported), 1 (reported but inadequate), or 2 (reported and adequate). A ratio between 0.75-1.00 indicated high quality, a ratio between 0.50-0.75 indicated intermediate quality, and a ration below 0.50 indicated low quality.

ROB was assessed using the Systematic Review Center for Laboratory Animal Experimentation (SYRCLE) tool that details six types of bias, including selection, performance, detection, attrition reporting, and other biases across ten domains. A judgment of "yes" indicated low ROB, and a judgement of "no" indicated high ROB. If insufficient details had been reported for proper assessment of ROB, then it would be judged as "unclear".

The kappa test was employed to assess interobserver consistency in both ARRIVE guidelines 2.0 and ROB evaluation. A high level of consistency was considered when the kappa value (κ) was ≥0.75, while a moderate level of consistency was indicated by κ values ranging from 0.40 to 0.75. A low level of consistency was observed when κ <0.40.

3.4 Data abstraction: Two researchers independently extracted data from the tables, graphs, or text. Discrepancies were resolved through consultation with a third researcher. If data were obtained from a graph, the average of the two values was calculated. In cases where the information in the included article was not clearly described, the authors were contacted via email, telephone, or other means to obtain missing or additional data.

3.5 Study characteristics: The data extracted from the analyzed studies included animal model characteristics (species and weight), number of animals used in the experiments, experimental design details, treatment regimen information (method and dosage of drug administration), timing of drug administration, treatment outcomes/results, and other study attributes, such as author and publication year.

3.6 Data synthesis

3.6.1 Parameters for evaluating the protective effects of Met against Dox-induced cardiotoxicity: (1) body weight and the relative heart weight ratio; (2) myocardial injury markers, including serum/plasma lactate dehydrogenase (LDH), creatine kinase-myocardial band (CK-MB), cardiac troponin I (cTnI), and aspartate aminotransferase (AST); (3) cardiac function indicators such as brain natriuretic peptide (BNP) and N-terminal B-type natriuretic peptide (NT-proBNP) measured in laboratory tests and echocardiogram results, including left ventricular ejection fraction (LVEF) and left ventricular fractional shortening (LVFS), cardiac output (CO), and stroke volume (SV); (4) electrocardiogram findings such as QT interval, corrected QT interval (QTc interval), and heart rate.

3.6.2 The mechanism underlying the protective effects exerted by Met against Dox-induced cardiotoxicity: (1) The oxidative stress hypothesis, including factors such as reactive oxygen

species (ROS), superoxide dismutase (SOD) activity, cyclooxygenase-2 (COX-2), nitrites and nitrates (NOx), nitric oxide (NO), nitric oxide synthase (iNOS), DNA load of iNOS level, malondialdehyde (MDA), thiobarbituric acid-reactive substances (TBARS), glutathione (GSH), glutathione peroxidase (GPx), DNA load concentration of Matrix metalloproteinase 2 (MMP2), catalase, and catalase/β-actin mRNA level. (2) The high-energy phosphate pool and alleviating energy starvation hypothesis involving adenosine triphosphate (ATP), blood glucose levels, mitochondrial swelling, CoA-SH, Acetyl-CoA, and adenosine 5'-monophosphate-activated protein kinase (AMPK). (3) Apoptosis and autophagy hypothesis, including cardiomyocyte apoptosis rate, B-cell lymphoma-2 (Bcl-2), cardiac caspase-3, cleaved caspase-3, cleaved/pro-caspase-3, Beclin-1, LC3B, LC3B-II, LC3-II/LC3-I, mammalian target of rapamycin (mTOR), P62, Smad3, TGFβ1 DNA load level, and TGFβ1. (4) The abnormal iron metabolism hypothesis, including the expression of ferritin heavy chain (FHC), total iron, and transferrin receptor (TfR) in cardiac tissue.

The primary efficacy outcomes included LDH, CK-MB, cTnI, and AST levels., which are commonly used markers of myocardial injury. Other parameters were considered as secondary outcomes in the meta-analysis. The effect size was measured using the standardized mean difference (SMD) for continuous variables. Due to the significant heterogeneity in the majority of the data, a random-effects model was employed for the meta-analysis.

3.7 Investigation of heterogeneity in primary efficacy outcomes: Heterogeneity was assessed using the I^2 statistic and was categorized as no (<25%), low (25–50%), moderate (50–75%), or high (>75%) heterogeneity [37,38]. H- and Cochrane Q-tests were conducted to assess heterogeneity among the included studies. A value of H=1 indicated no heterogeneity, H>1.5 suggested identifiable heterogeneity, and H<1.2 indicated homogeneity across all included studies. For the Q-test, a p-value <0.10 denoted significant heterogeneity among the included studies.

3.8 Revealing the origins of heterogeneity in the primary efficacy outcomes: If significant heterogeneity ($I^2 > 50\%$) was observed in the primary efficacy outcomes, sensitivity analyses were conducted to assess the robustness of the conclusions with respect to eligibility and analysis decisions. Sensitivity analysis was performed using the "leave-one-out" method. Additionally, subgroup analysis and meta-regression were employed to explore any expected substantial heterogeneity. Subgroup analyses were performed based on potential sources of heterogeneity, including metformin dosage, type of cardiotoxicity (acute/chronic), and animal species.

3.9 Assessment of reporting biases: The potential for publication bias was evaluated using funnel plots, Egger's linear regression analysis, and Begg's rank correlation test.

3.10 Statistics and statistical software: The continuous measurement data are presented as the mean ± standard deviation. Statistical significance was defined as p<0.05. The statistical software package Stata18 (Stata Corp., College Station, TX, USA) was used for meta-analysis.

4. The results: The results of this study will be reported following the guidelines specific to reporting of systematic reviews and meta-analyses of animal experiments.

2. Literature retrieval

2.1 Embase Session Results

Search number	Query	Results
#1	(cardiotoxicit* OR (('cardiac'/exp OR 'cardiac' OR 'cardiac'/exp OR cardiac) AND toxicit*)) AND [<1966-2023]/py	92226
#2	dox* AND [<1966-2023]/py	376888
#3	(dimethylbiguanidine OR dimethylguanylguanidine OR 'glucophage'/exp OR glucophage OR 'metformin'/exp OR metformin OR 'hydrochloride'/exp OR hydrochloride OR (('metformin'/exp OR metformin) AND hcl)) AND [<1966-2023]/py	235314
#4	(animal OR rodent OR rat OR mouse OR murine OR rabbit OR gerbil OR hamster OR pig OR cat OR dog OR primate OR 'guinea pig') AND [<1966-2023]/py	8388628
#5	#1 AND #2 AND #3 AND #4	139

....

2.2 PubMed Session Results

Search number	Query	Filters		Results
#1	(((((metformin [MeSH Terms]) OR (Dimethylbiguanidine)) OR (Dimethylguanylguanidine)) OR (Glucophage)) OR (Metformin Hydrochloride)) OR (Metformin HCl)	from 1900/1/1 - 2023/12/31	("metformin"[MeSH Terms] OR ("metformin"[MeSH Terms] OR "metformin"[All Fields] OR "dimethylbiguanidine"[All Fields]) OR ("metformin"[MeSH Terms] OR "metformin"[All Fields] OR "dimethylguanylguanidine"[All Fields]) OR ("metformin"[MeSH Terms] OR "metformin"[All Fields] OR "glucophage"[All Fields] OR "metformine"[All Fields] OR "metformin s"[All Fields] OR "metformins"[All Fields]) OR ("metformin"[MeSH Terms] OR "metformin"[All Fields] OR ("metformin"[All Fields] AND "hydrochloride"[All Fields]) OR "metformin hydrochloride"[All Fields]) OR ("metformin"[MeSH Terms] OR "metformin"[All Fields] OR ("metformin"[All Fields] AND "hcl"[All Fields]) OR "metformin hcl"[All Fields])) AND (1900/1/1:2023/12/31[pdat])	30,830
#2	"Doxorubicin"[MeSH Terms] OR (Doxorubicin [Title/Abstract]) OR (Dox [Text Word]) OR (Dox-induced) OR (doxorubicin-induced)	from 1900/1/1 - 2023/12/31	("Doxorubicin"[MeSH Terms] OR "Doxorubicin"[Title/Abstract] OR "Dox"[Text Word] OR "Dox-induced"[All Fields] OR "doxorubicin-induced"[All Fields]) AND (1900/1/1:2023/12/31[pdat])	88,555
#3	"Cardiotoxicity"[MeSH Terms] OR (Cardiotoxicit*) OR (Cardiac Toxicit*)	from 1900/1/1 - 2023/12/31	("Cardiotoxicity"[MeSH Terms] OR "cardiotoxicit*"[All Fields] OR (("cardiacs"[All Fields] OR "heart"[MeSH Terms] OR "heart"[All Fields] OR "cardiac"[All Fields]) AND "toxicit*"[All Fields])) AND (1900/1/1:2023/12/31[pdat])	54,762
#4	(animal) OR (rodent) OR (rat) OR (mouse) OR (murine) OR (rabbit) OR (gerbil) OR (hamster) OR	from 1900/1/1 - 2023/12/31	("animals"[MeSH Terms:noexp] OR "animal"[All Fields] OR ("rodent s"[All Fields] OR "rodentia"[MeSH Terms] OR "rodentia"[All Fields] OR "rodent"[All Fields] OR "rodents"[All Fields]) OR ("rats"[MeSH Terms] OR "rats"[All Fields] OR "rat"[All Fields]) OR ("mice"[MeSH Terms] OR "mice"[All Fields] OR "mouse"[All Fields] OR "mouse s"[All Fields] OR "mouses"[All Fields]) OR	27,297,906

	(pig) OR (cat) OR (dog) OR (primate) OR (guinea pig)		("mice"[MeSH Terms] OR "mice"[All Fields] OR "murine"[All Fields] OR "muridae"[MeSH Terms] OR "muridae"[All Fields] OR "murines"[All Fields] OR "murin"[All Fields]) OR ("rabbit s"[All Fields] OR "rabbits"[MeSH Terms] OR "rabbits"[All Fields] OR "rabbit"[All Fields]) OR ("gerbil s"[All Fields] OR "gerbillinae"[MeSH Terms] OR "gerbillinae"[All Fields] OR "gerbil"[All Fields] OR "gerbils"[All Fields]) OR ("cricetinae"[MeSH Terms] OR "cricetinae"[All Fields] OR "hamster"[All Fields] OR "hamsters"[All Fields] OR "hamster s"[All Fields]) OR ("swine"[MeSH Terms] OR "swine"[All Fields] OR "pig"[All Fields]) OR "cat"[All Fields] OR ("dogs"[MeSH Terms] OR "dogs"[All Fields] OR "dog"[All Fields]) OR ("primate s"[All Fields] OR "primates"[MeSH Terms] OR "primates"[All Fields] OR "primate"[All Fields]) OR ("guinea pigs"[MeSH Terms] OR ("guinea"[All Fields] AND "pigs"[All Fields]) OR "guinea pigs"[All Fields] OR ("guinea"[All Fields] AND "pig"[All Fields]) OR "guinea pig"[All Fields])) AND (1900/1/1:2023/12/31[pdat])	
#5	#1 AND #2 AND #3 AND #4	from 1900/1/1 - 2023/12/31	(("metformin"[MeSH Terms] OR ("metformin"[MeSH Terms] OR "metformin"[All Fields] OR "dimethylbiguanidine"[All Fields]) OR ("metformin"[MeSH Terms] OR "metformin"[All Fields] OR "dimethylguanylguanidine"[All Fields]) OR ("metformin"[MeSH Terms] OR "metformin"[All Fields] OR "glucophage"[All Fields] OR "metformine"[All Fields] OR "metformin s"[All Fields] OR "metformins"[All Fields]) OR ("metformin"[MeSH Terms] OR "metformin"[All Fields] OR ("metformin"[All Fields] AND "hydrochloride"[All Fields]) OR "metformin hydrochloride"[All Fields]) OR ("metformin"[MeSH Terms] OR "metformin"[All Fields] OR ("metformin"[All Fields] AND "hcl"[All Fields]) OR "metformin hcl"[All Fields])) AND 1900/01/01:2023/12/31[Date - Publication] AND (("Doxorubicin"[MeSH Terms] OR "Doxorubicin"[Title/Abstract] OR "Dox"[Text Word] OR "Dox-induced"[All Fields] OR "doxorubicin-induced"[All Fields]) AND 1900/01/01:2023/12/31[Date - Publication]) AND (("Cardiotoxicity"[MeSH Terms] OR "cardiotoxicit*"[All Fields] OR (("cardiacs"[All Fields] OR "heart"[MeSH Terms] OR "heart"[All Fields] OR "cardiac"[All Fields]) AND "toxicit*"[All Fields])) AND 1900/01/01:2023/12/31[Date - Publication]) AND (("animals"[MeSH Terms:noexp] OR "animal"[All Fields] OR ("rodent s"[All Fields] OR "rodentia"[MeSH Terms] OR "rodentia"[All Fields] OR "rodent"[All Fields] OR "rodents"[All Fields]) OR ("rats"[MeSH Terms] OR "rats"[All Fields] OR "rat"[All Fields]) OR ("mice"[MeSH Terms] OR "mice"[All Fields] OR "mouse"[All Fields] OR "mouse s"[All Fields] OR "mouses"[All Fields]) OR ("mice"[MeSH Terms] OR "mice"[All Fields] OR "murine"[All Fields] OR "muridae"[MeSH Terms] OR "muridae"[All Fields] OR "murines"[All Fields] OR "murin"[All Fields]) OR ("rabbit s"[All Fields] OR "rabbits"[MeSH Terms] OR "rabbits"[All Fields] OR "rabbit"[All Fields]) OR ("gerbil s"[All Fields] OR "gerbillinae"[MeSH Terms] OR "gerbillinae"[All Fields] OR "gerbil"[All Fields] OR "gerbils"[All Fields]) OR ("cricetinae"[MeSH Terms] OR "cricetinae"[All Fields] OR "hamster"[All Fields] OR "hamsters"[All Fields] OR "hamster s"[All Fields]) OR ("swine"[MeSH Terms] OR "swine"[All Fields] OR "pig"[All Fields]) OR "cat"[All Fields] OR ("dogs"[MeSH Terms] OR "dogs"[All Fields] OR "dog"[All Fields]) OR ("primate s"[All Fields] OR "primates"[MeSH Terms] OR "primates"[All Fields] OR "primate"[All Fields]) OR ("guinea pigs"[MeSH Terms] OR ("guinea"[All Fields] AND "pigs"[All Fields]) OR "guinea pigs"[All Fields] OR ("guinea"[All Fields] AND "pig"[All Fields]) OR "guinea pig"[All Fields])) AND 1900/01/01:2023/12/31[Date - Publication])) AND (1900/1/1:2023/12/31[pdat])	39

....

2.3 Web of Science Session Results

41 results from All Databases for:

(Cardiotoxicity OR Cardiotoxicit* OR Cardiac Toxicit*) AND (Doxorubicin OR Dox OR Dox-induced OR doxorubicin-induced) AND (Metformin OR dimethylebiguanidine OR Dimethylguanylguanidine OR glaucophane OR (Metformin Hydrochloride) OR (Metformin HCl)) AND (animal OR (in vivo) OR rodent OR rat OR mouse OR murine OR rabbit OR gerbil OR hamster OR pig OR cat OR dog OR primate OR (guinea pig)) (Topic)

Refined By: Document Types: Article. Click to remove this refine from your search.NOT Document Types: Review Article or Editorial Material or Case Report or Letter or Early Access or Correction. Click to remove this refine from your search.

| Timespan: 1900-01-01 to 2023-12-31 (Index Date)

....

2.4 Science Direct Session Results

Find articles with these terms:
(doxorubicin-induced OR Dox-induced) AND Cardiotoxicity AND Metformin
Year(s):
Year: 1900-2023 (from inception to 2023-12-31)

562 results

Article type:

Review articles (233)

Research articles (94)

Encyclopedia (8)

Book chapters (86)

Conference abstracts (30)

Case reports (1)

Correspondence (3)

Discussion (1)

Editorials (11)

Errata (1)

Mini reviews (1)

Practice guidelines (5)

Short communications (3)

Other (85)

....

2.5 Scopus Session Results

Organization of Provision

National University of Singapore, NUS-IP Library

Search scheme

(TITLE-ABS-KEY (cardiotoxicity OR (cardiotoxicit*) OR (cardiac AND toxicit*)) AND TITLE-ABS-KEY (doxorubicin OR doxorubicin OR dox OR dox-induced OR doxorubicin-

induced) AND TITLE-ABS-KEY (metformin OR dimethylbiguanidine OR dimethylguanylguanidine OR glucophage OR (metformin AND hydrochloride) OR (metformin AND hcl)) AND TITLE-ABS-KEY (animal OR rodent OR rat OR mouse OR murine OR rabbit OR gerbil OR hamster OR pig OR cat OR dog OR primate OR (guinea AND pig))) AND PUBYEAR < 2024

59

Document type

Article 32

Review 24

Editorial 2

Erratum 1

....

2.6 CNKI Session Results

中国知网

https://www.cnki.net/

主题：阿霉素 AND 心脏毒性 AND 二甲双胍

中英文扩展 OR 同义词扩展

发表时间：1915-01-01 —— 2023.12.31，

检索结果：总库 5 篇，其中学术期刊 3 篇，学位论文 1 篇、特色期刊 1 篇。

中文学术期刊论文：3 篇。

....

3. Literature included in this study

#1. Karim L.Z.A., Arif I.S., Al Saady F.A. Metabolomics of metformin's cardioprotective effect in acute doxorubicin induced-cardiotoxicity in rats. Systematic Reviews in Pharmacy. 2021;12(3):100-109.

#2. Arinno A, Maneechote C, Khuanjing T, Ongnok B, Prathumsap N, Chunchai T, Arunsak B, Kerdphoo S, Shinlapawittayatorn K, Chattipakorn SC, Chattipakorn N. Cardioprotective effects of melatonin and metformin against doxorubicin-induced cardiotoxicity in rats are through preserving mitochondrial function and dynamics. Biochem Pharmacol. 2021 Oct;192:114743. doi: 10.1016/j.bcp.2021.114743.

#3. Ajmal K., Rafique S., Afzal A., Sikandar A., Naeem U. Protective effects of metformin on doxorubicin-induced cardiotoxicity and its early detection. Medical Forum Monthly. 2020;31(9):185-189.

#4. Shaty M.H., Al-Ezzi M.I., Arif I.S., Basil D. Effect of metformin on inflammatory markers involved in cardiotoxicity induced by doxorubicin. Research Journal of Pharmacy and Technology. 2019;12(12):5815-5821.

#5. Shaty M.H., Arif I.S., Al-Ezzi M.I., Hanna D.B. Metformin attenuate fibrosis in both acute and chronic doxorubicin cardiotoxicity in rabbits. Journal of Pharmaceutical Sciences and Research. 2018; 10(6):1559-1565.

#6. Zilinyi R, Czompa A, Czegledi A, Gajtko A, Pituk D, Lekli I, Tosaki A. The Cardioprotective Effect of Metformin in Doxorubicin-Induced Cardiotoxicity: The Role of Autophagy. Molecules. 2018 May 15;23(5):1184. doi: 10.3390/molecules23051184.

#7. Aruna P., Gayathiri N.M. Cardioprotective activity of telmisartan, metformin and its combination against doxorubicin induced myocardial infarction in rat model. Research Journal of Pharmacy and Technology. 2018; 11(12):5293-5296. DOI: 10.5958/0974-360X.2018.00964.2

#8. Argun M., Üzüm K., Sönmez M.F., Özyurt A., Karabulut D., Soyersarıca Z., Çilenk K.T., Unalmış S., Pamukcu Ö., Baykan A., Narin F., Elmalı F., Narin N. Cardioprotective effect of metformin against doxorubicin cardiotoxicity in rats. Anatol J Cardiol. 2016 Apr;16(4):234-41. doi: 10.5152/akd.2015.6185.

#9. Sheta A, Elsakkar M, Hamza M, Solaiman A. Effect of metformin and sitagliptin on doxorubicin-induced cardiotoxicity in adult male albino rats. Hum Exp Toxicol. 2016 Nov;35(11):1227-1239. doi: 10.1177/0960327115627685.

#10. Mohamed EL Shabrawy Abdo, Afaf Sayed Osman, Omayma Anwar Khorshid, Lubna Omar El-Farouk and Mahmoud M Kamel. Comparative Study of the Protective Effect of

Metformin and Sitagliptin against Doxorubicin-Induced Cardiotoxicity in Rats. Clin Pharmacol Biopharm 2016, 6:3. DOI: 10.4172/2167-065X.1000174.

#11. Kelleni MT, Amin EF, Abdelrahman AM. Effect of Metformin and Sitagliptin on Doxorubicin-Induced Cardiotoxicity in Rats: Impact of Oxidative Stress, Inflammation, and Apoptosis. J Toxicol. 2015;2015:424813. doi: 10.1155/2015/424813.

#12. Ashour AE, Sayed-Ahmed MM, Abd-Allah AR, Korashy HM, Maayah ZH, Alkhalidi H, Mubarak M, Alhaider A. Metformin rescues the myocardium from doxorubicin-induced energy starvation and mitochondrial damage in rats. Oxid Med Cell Longev. 2012;2012:434195. doi: 10.1155/2012/434195.

#13. Mackay AD, Marchant ED, Munk DJ, Watt RK, Hansen JM, Thomson DM, Hancock CR. Multitissue analysis of exercise and metformin on doxorubicin-induced iron dysregulation. Am J Physiol Endocrinol Metab. 2019 May 1;316(5):E922-E930. doi: 10.1152/ajpendo.00140.2018.

#14. Ikewuchi JC, Ikewuchi CC, Ifeanacho MO, Jaja VS, Okezue EC, Jamabo CN, Adeku KA. Attenuation of doxorubicin-induced cardiotoxicity in Wistar rats by aqueous leaf-extracts of Chromolaena odorata and Tridax procumbens. J Ethnopharmacol. 2021 Jun 28;274:114004. doi: 10.1016/j.jep.2021.114004.

#15. Chen J, Zhang S, Pan G, Lin L, Liu D, Liu Z, Mei S, Zhang L, Hu Z, Chen J, Luo H, Wang Y, Xin Y, You Z. Modulatory effect of metformin on cardiotoxicity induced by doxorubicin via the MAPK and AMPK pathways. Life Sci. 2020 May 15;249:117498. doi: 10.1016/j.lfs.2020.117498.

#16. Satyam SM, Bairy LK, Shetty P, Sainath P, Bharati S, Ahmed AZ, Singh VK, Ashwal AJ. Metformin and Dapagliflozin Attenuate Doxorubicin-Induced Acute Cardiotoxicity in Wistar Rats: An Electrocardiographic, Biochemical, and Histopathological Approach. Cardiovasc Toxicol. 2023 Feb;23(2):107-119. doi: 10.1007/s12012-023-09784-8.

#17. Yi Y, Zhang H, Chen M, Chen B, Chen Y, Li P, Zhou H, Ma Z, Jiang H. Inhibition of multiple uptake transporters in cardiomyocytes/mitochondria alleviates doxorubicin-induced cardiotoxicity. Chem Biol Interact. 2023 Sep 1;382:110627. Doi: 10.1016/j.cbi.2023.110627.

#18. Alzokaky AA, Al-Karmalawy AA, Saleh MA, Abdo W, Farage AE, Belal A, Abourehab MAS, Antar SA. Metformin ameliorates doxorubicin-induced cardiotoxicity targeting HMGB1/TLR4/NLRP3 signaling pathway in mice. Life Sci. 2023 Mar 1;316:121390. doi: 10.1016/j.lfs.2023.121390.

#19. Wei J, Yang Q, Lin L, Zhu C, Wei J. [Metformin mitigates doxorubicin-induced cardiotoxicity via the AMPK pathway]. Nan Fang Yi Ke Da Xue Xue Bao. 2023 Oct 20;43(10):1682-1688. Chinese. Doi: 10.12122/j.issn.1673-4254.2023.10.05.

#20. Zhang S, Wei X, Zhang H, Wu Y, Jing J, Huang R, Zhou T, Hu J, Wu Y, Li Y, You Z. Doxorubicin downregulates autophagy to promote apoptosis-induced dilated cardiomyopathy via regulating the AMPK/mTOR pathway. Biomed Pharmacother. 2023 Jun;162:114691. Doi: 10.1016/j.biopha.2023.114691.

#21. Kong L, Gao J, Zhang J, Xu C, Sun N, Wei M, Su X. Metformin alleviates doxorubicin-induced cardiotoxicity by inhibiting endoplasmic reticulum stress via activation of SIRT1 signaling pathway. J Shanxi Med Univ. 2022, 53(10):1261-1267. Chinese. Doi: 10.12122/j.issn.1673-4254.2023.10.05.

4. The characteristics of the included studies (continue)

No.	Author (year)	Cumulative dose of Dox	Cumulative dose of Met	Experimental regimen	Results
#1	Abdul Karim LZ (2021)	20 mg/kg BW	2100 mg/kg BW	**Acute Dox induction group**: DOX (20 mg/kg, single dose, i.p.).<br>**Met + acute Dox group:** DOX (20mg/kg, single dose, i.p.), and Met (300 mg/kg/day, oral with gavage for 7 consecutive days starting five days prior to DOX treatment)	Met induced a metabolic alteration, including the promotion of glycogenolysis, glycolysis, amino acid utilization and antioxidation. Met improving energy metabolism and attenuating oxidative stress through suppression of serum MDA and increase the level of GSH as well as decrease fibrosis and structural changes.
#2	Arinno A (2021)	18 mg/kg BW	7500 mg/kg W	**Chronic Dox induction group:** DOX (3 mg/kg, intraperitoneal on day 0, 4, 8, 15, 22, and 29), and 0.9% Normal saline (oral with gavage every day).<br>**Met + chronic Dox induction group:** DOX (3 mg/kg, intraperitoneal on day 0, 4, 8, 15, 22, and 29), and Met (250 mg/kg/day, oral with gavage for 30 consecutive days).	DOX induced excessive oxidative stress, inflammation, autophagy, apoptosis, reduced mitochondrial function, dynamics balance, biogenesis, and bioenergetics leading to LV dysfunction. Treatment with Met exerted measures of cardio-protection via reducing oxidative stress, inflammation, autophagy, apoptosis, and improved mitochondrial function, dynamics balance, biogenesis, and bioenergetics in the Dox-treated rats.
#3	Ajmal K (2020)	12 mg/kg BW	2750 mg/kg BW	**Acute Dox induction group:** DOX (12 mg/kg, single dose, injected into marginal vein of rabbit's ear on Day 10).<br>**Met + acute Dox induction group:** Met (250 mg/kg/day, oral with gavage for 11 consecutive days), and DOX (12 mg/kg, single dose, injected on the Day 10).	DOX inflicted marked cardiac damage apparent by elevated serum biomarkers (LDH, CK-MB and cTnI) levels and necrosed cardiomyocytes on histological examination. Met pretreatment ensued in decreased serum levels of biomarkers and improved the histological grades of heart tissue.
#4	Shaty MH (2019)	16 mg/kg BW	4200 mg/kg BW	**Acut**e **Dox induction group:** DOX (16mg/kg, single dose, i.p.).<br>**Met** + **acute Dox induction group:** DOX (16mg/kg, single dose, i.p.) and Met (300mg/kg/day, oral with gavage daily for 14 consecutive days, starting three days prior to Dox therapy).<br>**Chronic Dox induction group:** DOX (4mg/kg, twice a week for two weeks, i.p.).	Met treatment significantly ($p<0.05$) decreased the MMP2, TNFα, iNOS level in addition to improve the histological change of cardiac tissues produced by acute and chronic DOX induction toxicity.

				**Met + chronic DOX induction group**: DOX (4mg/kg, twice a week for two weeks, i.p.), and Met (300 mg/kg/day, oral with gavage daily for 14 consecutive days, starting three days prior to DOX therapy).	
#5	Shaty MH (2018)	16 mg/kg BW	4200 mg/kg BW	**Acute DOX induction group:** DOX (16 mg/ kg, single dose, i.p.).<br>**Met + acute DOX induction group:** DOX (16 mg/kg, single dose, i.p.), and Met (300 mg/kg/day, oral with gavage for 14 consecutive days, starting three days prior to Dox treatment).<br>**Chronic DOX induction group:** DOX (4mg/kg, intraperitoneal twice a week).<br>**Met + chronic DOX group**: Dox (4 mg/kg, intraperitoneal twice a week) and Met (300 mg/kg/day, oral with gavage for 14 consecutive days, starting three days prior to Dox treatment).	Pretreatment with Met significantly ($p<0.05$) decreased the level of serum troponin I, SMAD3 and TGF-β1 in both MET +acute DOX and Met +chronic DOX group in compare with the acute DOX and chronic DOX group, in addition to significantly decreased collagen fiber production.
#6	Zilinyi R (2018)	18 mg/kg BW	3500 mg/kg BW	**Chronic DOX induction group:** DOX (3 mg/kg, intraperitoneal every second day), and oral water with gavage.<br>**Met + chronic DOX induction group:** DOX (3 mg/kg/day, intraperitoneal every second day), and Met (250 mg/kg/day, oral with gavage each day).	Treatment with Met conferred increased cardiac protection against the development of cardiotoxicity manifested by a significant decrease in serum TnT and cardiac MDA levels, and remarkable improvement in heart function in connection with histopathological features. Furthermore, by focusing on the contribution of autophagic proteins, it was found that Met normalized autophagy, which may help cardiomyocytes survive DOX-induced toxicity.
#7	Aruna P (2018)	30 mg/kg BW	2250 mg/kg BW	**Acute Dox induction group:** DOX (15 mg/kg, intraperitoneal on 14th, 15th day, i.p.).<br>**Low dose Met + acute DOX induction group:** Met (150mg/kg/day, oral with gavage for 15 consecutive days), and DOX (15mg/kg, intraperitoneal one hour after Met oral with gavage on 14th, 15th day).	The biochemical parameters, TnT shown to be negative for low-dose Met treated groups and positive for DOX group (negative control). LDH and CK-MB decreased for drug treated group then the negative control.

				**High dose Met + acute DOX induction group:** Met (300mg/kg/day, oral with gavage for 15 consecutive days), and DOX (15mg/kg, intraperitoneal one hour after Met administration on 14th, 15th day).	
#8	Argun M (2016)	16 mg/kg BW	3500 mg/kg BW	**Chronic DOX induction group:** DOX (4 mg/kg, intraperitoneal twice a week). **Met + chronic Dox induction group:** DOX (4 mg/kg, intraperitoneal twice a week), and Met (250 mg/kg/day, oral with gavage for 14 consecutive days, starting three days prior to DOX treatment).	DOX treatment caused significant deterioration in left ventricular functions by echocardiography, histological heart tissue damage, and increase in cardiomyocyte apoptosis. DOX + Met group showed protection in left ventricular function, elimination of histopathologic change, and reduced of cardiomyocyte apoptosis.
#9	Sheta A (2016)	20 mg/kg BW	3500 mg/kg BW	**Chronic DOX induction group:** DOX (20 mg/kg, intraperitoneal on Day 2 and 4). **Met + chronic DOX induction group:** DOX (20 mg/kg, intraperitoneal on Day 2 and 4), and Met (500 mg/kg, oral with gavage for 7 consecutive days beginning on Day 1).	Heart tissue was measured cardiac reduced GSH, TBARS, and TNFa. Serum CK and lactate LDH were also measured. Intoxication of DOX was associated with a significant elevation in serum CK-MB and LDH, reduction in cardiac GSH, and increased TBARS and TNFa. Administration of Met to DOX-intoxicated rats suppressed serum CK-MB and LDH. Moreover, cardiac GSH was elevated with decreased TBARS and TNFa. Met caused inhibition of caspase 3 and upregulation of Bcl-2 expression in DOX-intoxicated animals.
#10	Shabrawy Abdo MEL (2016)	15 mg/kg BW	3150 mg/kg BW	**Chronic DOX induction group:** DOX (1.0 mg/kg, 5 injections per week, i.p.). **Met + chronic DOX induction group:** DOX (1.0 mg/kg, 5 injections per week, i.p.), and Met (150 mg/kg/day, oral with gavage for 3 consecutive weeks).	DOX induced marked cardiotoxicity evidenced by significant deterioration in body weight, systolic BP and HR, elevation of ST segment, prolongation of QT interval, elevation in the serum level of CK-MB and LDH, blood glucose and cardiac MDA level and reduced in vitro cardiac contractility in response to isoprenaline. Administration of either Met with DOX resulted in significant improvement in all tested parameters compared with DOX treated rats.
#11	Kelleni MT (2015)	15 mg/kg BW	1050 mg/kg BW	**Acute Dox induction group:** DOX (a single dose of 15mg/kg, intraperitoneal at Day 5). **Met + acute DOX induction group:** DOX (a single dose of 15mg/kg, intraperitoneal at Day 5), and Met (250mg/kg/day, oral with gavage for 7 consecutive days).	Pretreatment with Met produced significant ($P<0.05$) cardiac protection manifested by a significant decrease in serum levels of LDH and CK-MB enzymes and cardiac MDA and total nitrites and nitrates levels, a significant increase in cardiac SOD activity, and remarkable improvement in the histopathological features as well as a significant reduction in the immunohistochemical expression of COX-2, iNOS, and caspase-3 enzymes as compared to DOX group.
#12	Ashour AE	18 mg/kg BW	550 mg/kg BW	**Chronic DOX induction group:** DOX (3 mg/kg, intraperitoneal every other day over	DOX treatment significantly increased serum levels of LDH and CK-MB, and induced expression of hypertrophic gene markers. DOX also caused marked

	(2012)			a period of 11 days).<br>**Low dose Met + chronic DOX induction group:** DOX (3 mg/kg, intraperitoneal every other day over a period of 11 days), and Met (50 mg/kg/day, oral with gavage for 11 consecutive days).<br>**High dose Met + chronic DOX induction group:** DOX (3 mg/kg, intraperitoneal every other day over a period of 11 days), and Met (500 mg/kg/day, oral with gavage for 11 consecutive days).	decreases in the cardiac levels of glutathione, CoA-SH and ATP, and mRNA expression of catalase and NQO-1.
#13	Mackay AD (2019)	15 mg/kg BW	2500 mg/kg BW	**Acute DOX induction group:** DOX (15 mg/kg, single dose, i.p.).<br>**Acute Met + Acute DOX induction group:** Met (500 mg/kg/day, oral with gavage, beginning 2 days before DOX treatment and continuing until euthanasia).	The cellular response to DOX is protective against oxidative stress by reducing iron availability. DOX increased iron storage capacity through elevated ferritin levels in liver, heart, and skeletal muscle. DOX reduced iron transport capacity through reduced transferrin receptor levels in heart and skeletal muscle. Met treatment had protective effects in the liver through reduced transferrin receptor levels.
#14	Ikewuchi JC (2021)	15 mg/kg BW	3500 mg/kg BW	**Acute DOX induction group:** DOX (15 mg/kg, single dose, intra-peritoneal on Day 12).<br>**Met + Acute DOX induction group:** DOX (15 mg/kg, single dose, intraperitoneal on Day 12), and Met **(**250 mg/kg/day, oral with gavage).	Pre-treatment with Met protected the heart, by preventing DOX-induced adverse alterations in plasma markers of cardiac functions/integrity (LDH, CK-MB, etc), cardiac markers of oxidative stress (MDA, reduced GSH, etc), and so on.
#15	Chen J (2020)	20 mg/kg BW	725, 2900, 11600 mg/kg BW	**Chronic DOX induction group:** DOX (5 mg/kg, intraperitoneal on days 7, 14, 21, and day 28**).**<br>**Met + chronic DOX induction group:** DOX (5 mg/kg, intraperitoneal on days 7, 14, 21, and day 28), and Met (25 mg/kg in low dose group 1, 100 mg/kg in low dose group 2, and 400 mg/kg in high dose group; oral with gavage**).**	The body weight loss trend of Met treatment group 1 rats and Met treatment group 2 rats were relieved compared with the DOX group ($p < 0.05$). Compared with the DOX group, Met treatment could significantly reduce the serum LDH and CK-MB level, the results were extraordinary in the Met treatment group ($p < 0.05$). Treatment with Met mitigated the impact towards MDA, and GSH levels induced by DOX. Met treatment significantly increased DOX-induced decrease of the LVEF, LVFS, etc and the protection was dose-related.
#16	Satyam	20 mg/kg	2000, 1440	**Acute DOX induction group:** DOX (20	CK-MB, AST, and glucose were significantly increased in DOX control rats.

	SM (2023)		mg/kg BW	mg/kg, single dose, intraperitoneal on 7th day).<br>**Met + acute Dox induction group:** Met (250 mg/kg/day in **medium-dose Met group** and 180 mg/kg/day in **low-dose Met group**, oral with gavage for 8 consecutive days), and Dox (20 mg/kg, single dose, intraperitoneal on Day 7).	There was a significant reversal of DOX-induced hyperglycemia in the rats treated with Met 250 mg/kg compared to DOX control rats. Both Met (180 mg/kg and 250 mg/kg) significantly altered DOX-induced ECG changes and reduced the levels of cardiac injury biomarkers CK-MB and AST compared to DOX control rats. Met protected the cellular architecture of the myocardium from DOX-induced myocardial injury. Current study revealed that both Met at the FDA-recommended antidiabetic doses mitigated DOX-induced acute cardiotoxicity in Wistar rats. The obtained data have opened the perspective to perform chronic studies and then to clinical studies to precisely consider Met as potential chemoprotection in the combination of chemotherapy with DOX to limit its cardiotoxicity.
#17	Yi Y (2023)	24 mg/kg	1050, 2100, 4200, 5250, 10500 mg/kg BW	**Chronic Dox induction group:** DOX (8 mg/kg, intravenous injection at day 7, 14 and 21).<br>**Met + chronic DOX induction group:** DOX (8 mg/kg, intravenous injection one hour after Met administration at day 7, 14 and 21), and Met (50 mg/kg/day in **low-dose group 1**, 100 mg/kg/day in **low-dose group 2**, 200 mg/kg/day in **medium-dose Met group 1**, oral with gavage for three consecutive weeks; and 250 mg/kg/day in **medium-dose group 2**, 500 mg/kg/day in **high-dose group**, intravenous injection for three consecutive weeks, Met dissolved in water) were administered by oral gavage every day for three weeks.	OCTN1/OCTN2/PMAT (organic cation/carnitine transporter 1/2 or plasma membrane monoamine transporter), especially OCTN2, played crucial roles in DOX uptake in cardiomyocytes, while OCTN2 and OCTN1 contributed to DOX transmembrane transport in mitochondria. Metformin (1–100 μM) concentration-dependently reduced DOX (5 μM for accumulation, 500 nM for cytotoxicity) concentration and toxicity in cardiomyocytes/mitochondria via inhibition of OCTN1-, OCTN2- and PMAT-mediated DOX uptake but did not affect its efflux. Furthermore, metformin (iv: 250 and 500 mg/kg or ig: 50, 100 and 200 mg/kg) could dose-dependently reduce DOX (8 mg/kg) accumulation in mouse myocardium and attenuated its cardiotoxicity. In addition, metformin (1–100 μM) did not impair DOX efficacy in breast cancer or leukemia cells. This study clarified the role of multiple transporters, especially OCTN2, in DOX uptake in cardiomyocytes/mitochondria; metformin alleviated DOX-induced cardiotoxicity without compromising its antitumor efficacy by selective inhibition of multiple transporters mediated DOX accumulation in myocardium/mitochondria.
#18	Alzokaky AA (2023)	15 mg/kg	2800 mg/kg BW	**Acute DOX induction group:** DOX (15 mg/kg, single dose, i.p.).<br>**Met + acute DOX induction group:** DOX (15 mg/kg, single dose, i.p.), and Met (200 mg/kg/day, started oral with gavage one week before the intraperitoneal of DOX for 14 consecutive days.	Heart weight, cardiac cTnT, CK-MB levels, MDA, and NO contents all increased significantly in the DOX group to the control normal group. Conversely, there was a substantial decline in SOD and GSH. DOX group depicts a high expression of TLR4, HMGB1, and caspase 3. Immunohistochemical staining revealed an increase in NLRP3 inflammasome and NF-κB expressions alongside histopathological modifications. Met dramatically decreased DOX-induced abnormal cardiac weight, CK-MB, and

					cTnT while maintaining the tissues' histological integrity. Inflammatory biomarkers, including HMGB1, TLR4, NF-κB, inflammasome, and caspase 3 were reduced after Met therapy. Furthermore, molecular docking studies suggested the antagonistic activity of Met towards HMGB1, NF-κB, and caspase 3 target receptors. Met is a desirable strategy for improving cardiotoxicity produced by DOX by inhibiting the HMGB1/NF-κB inflammatory pathway, thus preserving heart function.
#19	Wei J (2023)	20 mg/kg	1200 mg/kg BW	**Acute DOX induction group:** 20 mg/kg (single dose), intraperitoneal on Day 3.<br>**Medium-dose Met + acute DOX induction groups:** Met (200 mg/kg/day, oral with gavage for 6 consecutive days), and DOX (20 mg/kg, single dose, intraperitoneal on Day 3).<br>Each of the above two groups is divided into groups WT group and AKO group depending on the experimental animal (wild-type and C57BL/6 mice with AMPKα2 gene knockout mice).	CK-MB, LDH and BNP levels increased and EF and FS decreased significantly in the control group after chemotherapy ($P<0.05$). In the test group, CK-MB, LDH and BNP levels were significantly lowered after the combined treatment ($P<0.05$), while EF and FS did not undergo obvious changes ($P>0.05$). CK-MB, LDH and BNP levels were lower and EF and FS were higher significantly in the test group than in the control group after the treatment ($P<0.05$). DOX treatment reduced FS in both wild-type and AKO mice, but the reduction was less obvious in AKO group ($P<0.05$). The combined treatment restored FS in wild-type mice ($P<0.05$) but not in AKO mice. DOX significantly increased LDH and cTnI levels in both wild- type and AKO mice, but with smaller increments in the latter ($P<0.05$); The combined treatment with Met reduced DOX-induced elevation of LDH and cTnI levels in the wild-type mice ($P<0.05$) but not in AKO group ($P>0.05$). DOX increased myocardial cell apoptosis in both WT mice ($P<0.01$) but less strongly in AKO mice ($P<0.05$). Chemotherapy with DOX causes cardiotoxicity, which can be mitigated by combined treatment with Met possibly through a mechanism involving the AMPK pathway.
#20	Zhang S (2023)	20 mg/kg	2800 mg/kg BW	**Chronic DOX induction group:** intravenous injection with 5 mg/kg of DOX on days 1, 7, 14, and 21;<br>**Low-dose Met + chronic DOX induction group:** given Met orally at a dose of 100 mg/kg 2 h before 5 mg/kg DOX treatment on days 1, 7, 14 and 21, and given Met orally at a dose of 100 mg/kg daily for the remaining 28 experimental days.	In vivo, we used Met which is an AMPK activator to protect cardiac tissue to alleviate DOX-induced dilated cardiomyopathy. In this study, Met significantly attenuated the oxidative stress response of myocardial tissue caused by DOX and activated cardiomyocyte autophagy to maintain cardiomyocyte energy metabolism and reduce cardiomyocyte apoptosis by downregulating mTOR activity. Overall, our study revealed the role of autophagy and apoptosis in DOX-induced dilated cardiomyopathy and demonstrated the potential role of regulation of the AMPK/mTOR axis in the treatment of DOX-induced dilated cardiomyopathy.
#21	Kong L	15 mg/kg	350, 700	**Acute DOX induction group:** DOX (15	LVEF, LVFS and heart weight/tibial length (HW/TL) were significantly

	(2022)		mg/kg BW	mg/kg, single dose i.p.).<br>**Met + acute DOX induction group:** Met (50 mg/kg/day in **Low-dose Met group 1**, and 100 mg/kg/day in **Low-dose Met group 2**, oral with gavage for 7 consecutive days), and Dox (15 mg/kg, single dose, i.p.).	decreased in DOX group ($p < 0.01$), the myocardial fiber was swollen and vacuolization, serum LDH was increased ($P < 0.01$), the protein levels of cleaved Caspase-3, cytochrome C, PERK, p-eIF2α and Ac-Foxo1 and the apoptotic rate were increased ($P < 0.01$)，and the protein levels of GRP78 and SIRT1 were decreased ($P < 0.01$). Compared with DOX group，the cardiac function and structure were improved in low dose group, LVEF, LVFS and HW/TL were increased ($P < 0.05$), the myocardial fiber changes were alleviated, serum LDH was decreased ($P < 0.05$), the protein levels of cleaved Caspase-3, cytochrome C, PERK, p-eIF2α and Ac-Foxo1 and the apoptotic rate were decreased ($P < 0.05$), and the protein levels of GRP78 and SIRT1 were increased($P < 0.05$) . Compared with DOX group，the cardiac function and structure were improved in high dose group, LVEF, LVFS and HW/TL were increased($P < 0.01$), the myocardial fiber changes were alleviated, serum LDH was decreased ($P < 0.01$), the protein levels of cleaved Caspase-3, cytochrome C, PERK, p-eIF2α and Ac-Foxo1 and the apoptotic rate were decreased ($P < 0.01$), and the protein levels of GRP78 and SIRT1 were increased ($P < 0.01$). Metformin could inhibit the endoplasmic reticulum stress and improve the DOX-induced cardiotoxicity by activating SIRT1 signaling .

BW = body weight. NSS, normal saline solution. i.p., intraperitoneal injection. p.o. = per os. Dox = Doxorubicin. Met = metformin.

5. Quality assessment according to the Animal Research: Reporting In Vivo Experiment (ARRIVE) guidelines 2.0

No.	Studies	ARRIVE Essential 10										Recommended Set											quality score	Max score	Ratio	κ
		1	2	3	4	5	6	7	8	9	10	11	12	13	14	15	16	17	18	19	20	21				
#1	Abdul KLZ (2021)	2	1	2	1	1	2	2	1	2	2	2	2	2	0	2	0	1	1	1	1	0	28	42	.667	.845
#2	Arinno A (2021)	2	1	2	1	1	2	2	2	2	2	2	2	2	2	2	1	2	1	1	1	2	35	42	.833	1.00
#3	Ajmal K (2020)	2	1	2	1	1	2	2	1	2	2	2	2	2	2	1	1	1	1	1	1	2	32	42	.762	.725
#4	Shaty MH (2019)	2	1	2	1	1	2	2	2	2	2	2	2	2	0	2	1	1	1	1	1	0	30	42	.714	.916
#5	Shaty MH (2018)	2	1	2	1	1	2	2	2	2	2	2	2	2	0	2	1	1	1	1	1	0	30	42	.714	.916
#6	Zilinyi R (2018)	2	1	1	1	1	2	2	1	2	2	2	2	2	2	2	1	2	1	1	2	2	34	42	.810	.788
#7	Aruna P (2018)	2	1	2	0	1	2	0	2	2	2	2	2	2	2	2	1	1	1	1	1	0	29	42	.690	1.00
#8	Argun M (2016)	2	1	2	1	2	2	2	1	2	2	2	2	2	1	2	1	2	1	1	1	1	33	42	.786	.545
#9	Sheta A (2016)	2	1	2	1	1	2	2	2	2	2	2	2	2	2	2	1	1	2	1	1	2	35	42	.833	.897
#10	Shabrawy AM (2016)	2	1	2	0	1	2	2	1	2	2	2	2	2	2	2	1	1	2	1	1	2	33	42	.786	.622
#11	Kelleni MT (2015)	2	1	1	1	1	2	2	1	2	2	2	2	2	2	2	1	1	1	1	1	1	31	42	.738	.484
#12	Ashour AE (2012)	2	1	2	1	1	2	2	2	2	2	2	2	2	2	2	1	1	1	1	1	2	34	42	.810	.901
#13	Mackay AD (2019)	2	1	2	1	1	2	2	1	2	2	2	2	2	2	2	2	1	1	1	1	2	34	42	.810	.541
#14	Ikewuchi JC (2021)	2	2	2	0	1	2	2	2	2	2	2	2	2	0	2	1	1	0	1	2	2	32	42	.762	.736
#15	Chen J (2020)	2	1	2	0	1	2	2	2	2	2	2	2	2	2	2	2	2	0	1	1	2	34	42	.810	.899
#16	Satyam SM (2023)	2	1	2	1	1	2	2	2	2	2	2	2	2	2	2	2	1	1	1	1	2	35	42	.833	.897
#17	Yi Y (2023)	2	1	2	1	1	2	2	2	2	2	2	2	2	2	2	2	2	2	1	2	2	38	42	.905	.136
#18	Alzokaky AA (2023)	2	1	2	1	1	2	2	2	2	2	2	2	2	2	2	2	1	2	1	1	0	34	42	.810	1.00
#19	Wei J (2023)	2	1	2	1	1	2	2	2	2	1	2	2	2	2	2	2	2	2	1	1	1	35	42	.833	.889
#20	Zhang S (2023)	2	2	2	1	1	2	2	2	2	2	2	2	2	2	2	2	1	1	1	2	2	37	42	.881	.877
#21	Kong L (2022)	2	1	2	1	1	2	2	2	2	2	2	2	2	2	0	2	1	2	1	1	1	33	42	.786	.903
Quality score		42	23	40	17	22	42	40	35	42	41	42	42	42	33	39	28	27	25	21	25	28	696			
Maximum score		42	42	42	42	42	42	42	42	42	42	42	42	42	42	42	42	42	42	42	42	42		882		
Ratio of Q/M		1	.55	.95	.40	.52	1	.95	.83	1	.98	1	1	1	.79	.93	.67	.64	.60	.5	.60	.67	.79		.789	

κappa	/	/	-.11	1	1	/	1	/	/	/	/	/	/	.34	0.3	.42	.43	.84	/	1	.84				.820

Note: 1: study design; 2: sample size; 3: inclusion and exclusion criteria; 4: randomization; 5: blinding; 6: outcome measures; 7: statistical methods; 8: experimental animals; 9: experimental procedures; 10: results. 11: abstract; 12: background; 13: objectives; 14: ethical statement; 15: housing and husbandry; 16: animal care and monitoring; 17: interpretation/scientific implications; 18: generalizability/translation; 19: protocol registration; 20: data access; 21: declaration of interests.

6. Risk of Bias (ROB) assessing with SYRCLE's RoB tool for each experimental animal studies.

No.	Author (year)	Selection bias			Performance bias		Detection bias		Attrition bias	Reporting bias	Other	κ	p
		Sequence generation	Baseline characteristics	Allocation concealment	Random housing	Blinding	Random outcome assessment	Blinding	Incomplete outcome data	Selective outcome reporting	Other sources of bias		
#1	Abdul KLZ (2021)	U	U	U	Y	U	Y	U	Y	Y	Y	1.00	.002
#2	Arinno A (2021)	U	U	U	Y	U	Y	U	U	U	Y	.600	.038
#3	Ajmal K (2020)	U	Y	U	Y	U	Y	U	Y	Y	Y	1.00	.002
#4	Shaty MH (2019)	U	U	U	Y	U	Y	U	Y	Y	Y	1.00	.002
#5	Shaty MH (2018)	U	U	U	Y	U	Y	U	Y	Y	Y	1.00	.002
#6	Zilinyi R (2018)	U	U	U	Y	U	Y	U	U	U	Y	.600	.038
#7	Aruna P (2018)	U	U	U	U	U	U	U	Y	Y	Y	.583	.065
#8	Argun M (2016)	U	U	U	Y	U	Y	Y	Y	Y	Y	1.00	.002
#9	Sheta A (2016)	U	U	U	Y	U	Y	U	Y	Y	Y	1.00	.002
#10	Shabrawy AM (2016)	U	Y	U	U	U	U	U	Y	Y	Y	.583	.065
#11	Kelleni MT (2015)	U	U	U	Y	U	Y	U	Y	Y	Y	1.00	.002
#12	Ashour AE (2012)	U	U	U	Y	U	Y	U	Y	Y	Y	1.00	.002
#13	Mackay AD (2019)	U	Y	U	Y	U	Y	U	U	U	Y	.615	.035
#14	Ikewuchi JC (2021)	U	U	U	U	U	U	U	Y	Y	Y	.583	.065
#15	Chen J (2020)	U	U	U	Y	U	Y	U	Y	Y	Y	1.00	.002
#16	Satyam SM (2023)	U	Y	U	Y	U	Y	U	Y	Y	Y	.800	.01
#17	Yi Y (2023)	U	U	U	Y	U	Y	U	Y	Y	Y	1.00	.002
#18	Alzokaky AA (2023)	U	U	U	Y	U	Y	U	Y	Y	Y	1.00	.002
#19	Wei J (2023)	U	U	U	Y	U	Y	U	U	Y	Y	.800	.01
#20	Zhang S (2023)	U	U	U	Y	U	Y	U	Y	Y	Y	1.00	.002
#21	Kong L (2022)	U	U	U	Y	U	Y	U	Y	Y	Y	1.00	.002
	κ	-	0.859	-	-	-	-	1.000	-	-	-	.867	.000
	p	-	.000	-	-	-	-	.000	-	-	-	.000	

SYRCLE = Systematic Review Center for Laboratory animal Experimentation. Y: low risk of bias; U: unclear; N: high risk of bias.

7. Subgroup- and meta-regression analysis for serum lactate dehydrogenase (LDH)

7.1 Subgroup analysis for serum LDH

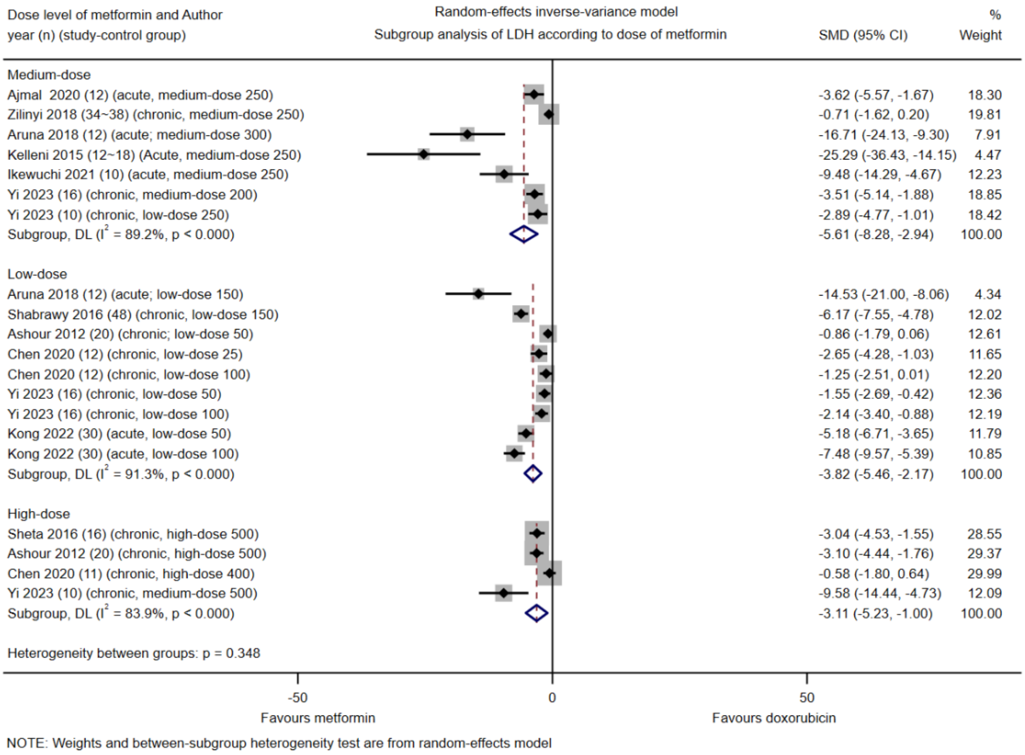

Fig. S1 Subgroup analysis according to dose of metformin for serum LDH

Note: Subgroup analysis disclosed that dose of metformin was not source of heterogeneity for serum lactate dehydrogenase.

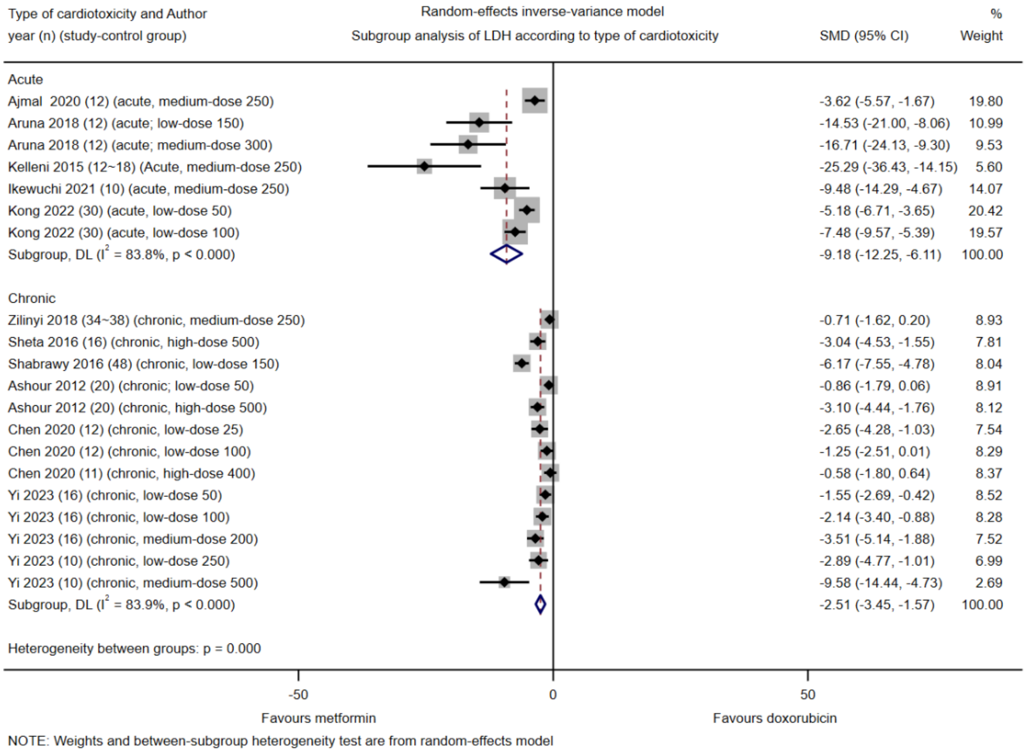

Fig. S2 Subgroup analysis according to type of cardiotoxicity for serum LDH

Note: Subgroup analysis disclosed that type of cardiotoxicity was not source of heterogeneity for LDH.

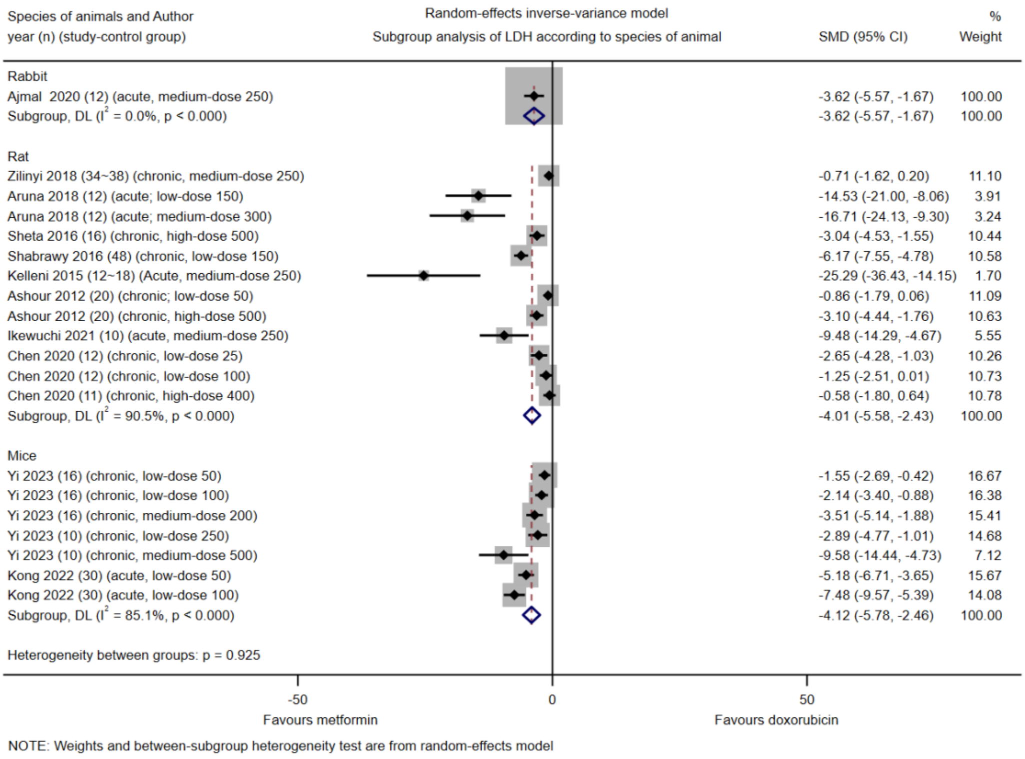

Fig. S3 Subgroup analysis according to species of animal for serum LDH

Notes: Subgroup analysis disclosed that species of animal was not source of heterogeneity for LDH.

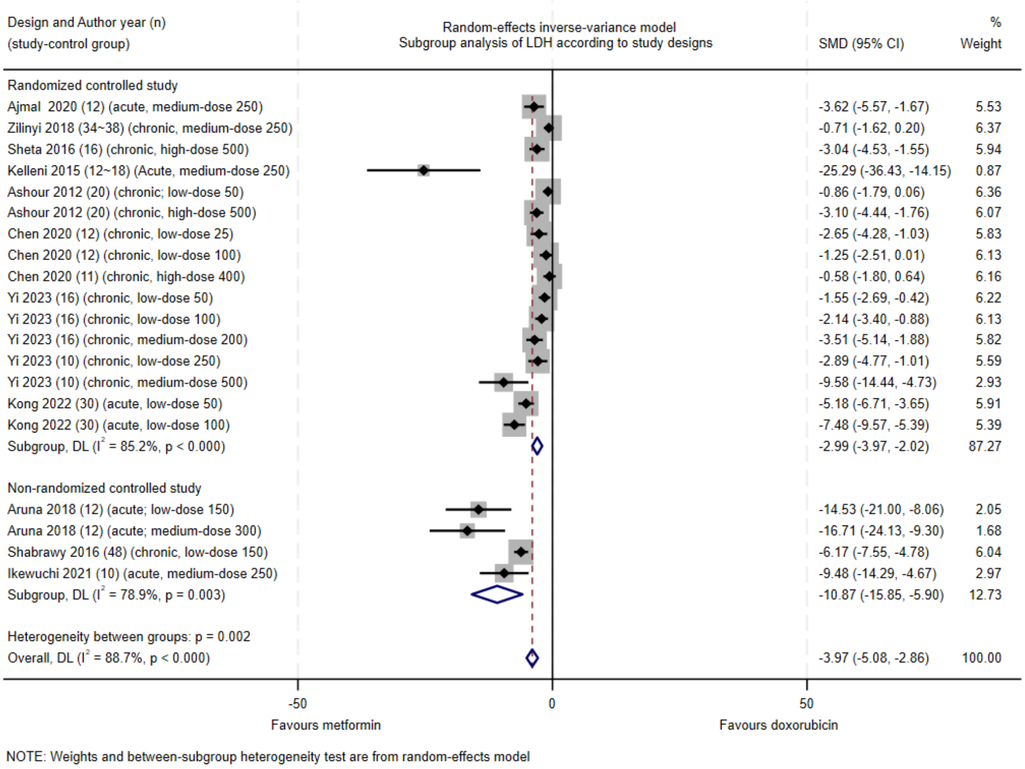

Fig. S4 Subgroup analysis according to study designs for serum LDH

Notes: Subgroup analysis disclosed that study designs was not source of heterogeneity for LDH.

7.2 Meta-regression analysis for serum LDH

Meta-regression	Number of obs = 20
REML estimate of between-study variance	tau2 = 5.51
% residual variation due to heterogeneity	I-squared_res = 83.84%
Proportion of between-study variance explained	Adj R-squared = 58.04%
With Knapp-Hartung modification	

_ES	Coefficient	Std. err.	t	P>|t|	[95% conf.	interval]
kindofcardioto~y	-5.661665	1.718477	-3.29	0.004	-9.272052	-2.051279
_cons	3.019511	2.271162	1.33	0.200	-1.752023	7.791046

Meta-regression	Number of obs = 20
REML estimate of between-study variance	tau2 = 1.981
% residual variation due to heterogeneity	I-squared_res = 73.91%
Proportion of between-study variance explained	Adj R-squared = 84.91%
Joint test for all covariates	Model F(4,15) = 5.36
With Knapp-Hartung modification	Prob > F = 0.0069

_ES	Coefficient	Std. err.	t	P>|t|	[95% conf.	interval]
doselevelofmet	-.5503731	.707196	-0.78	0.449	-2.057726	.9569794
kindofcardiotoxicity	-4.389983	1.602186	-2.74	0.015	-7.804961	-.9750056
kindofanimal	.0461159	1.140932	0.04	0.968	-2.385723	2.477955
studydesigns	5.178366	1.992629	2.60	0.020	.9311778	9.425554
_cons	-1.944534	2.77622	-0.70	0.494	-7.861907	3.972838

Note: The results of meta-regression analysis implied that type of cardiotoxicity contributed to 83.84% of heterogeneity for LDH (tau^2 = 5.51, residual I^2 = 83.84%, adjusted R^2 = 58.04%, p = 0.004).

8. Subgroup- and sensitivity analysis for serum creatine kinase-myocardial band (CK-MB)

8.1 Subgroup analysis for serum CK-MB

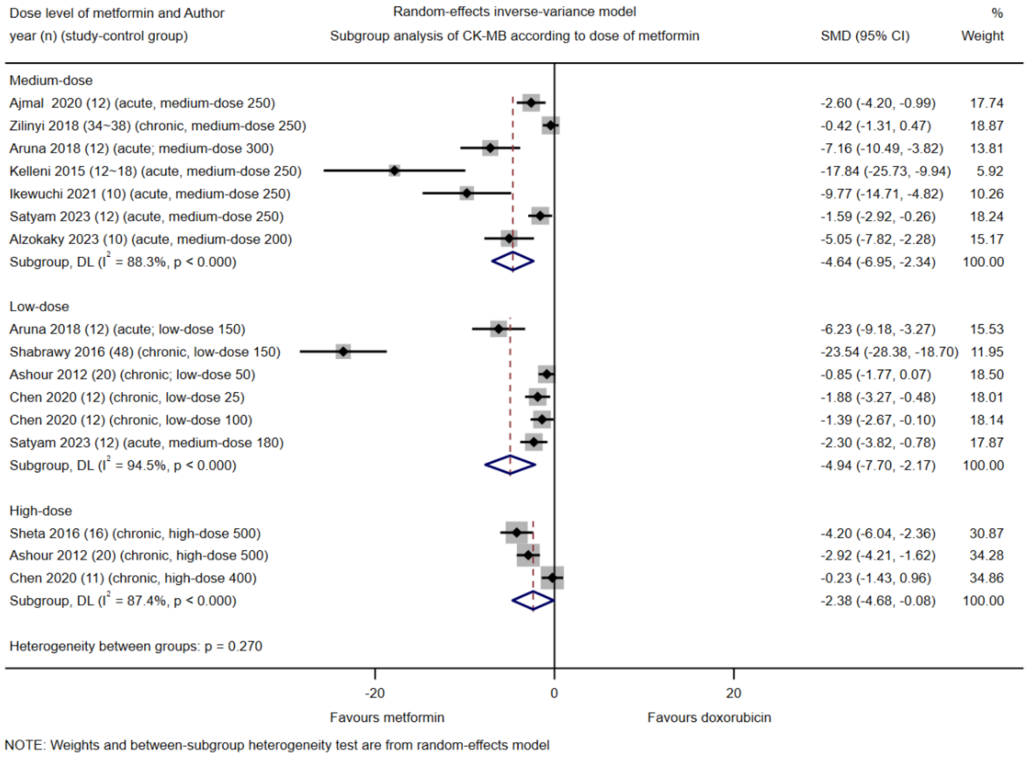

Fig. S5 Subgroup analysis according to dose of metformin for serum CK-MB

Note: Subgroup analysis discovered that dose of metformin did not contribute to the heterogeneity for serum CK-MB.

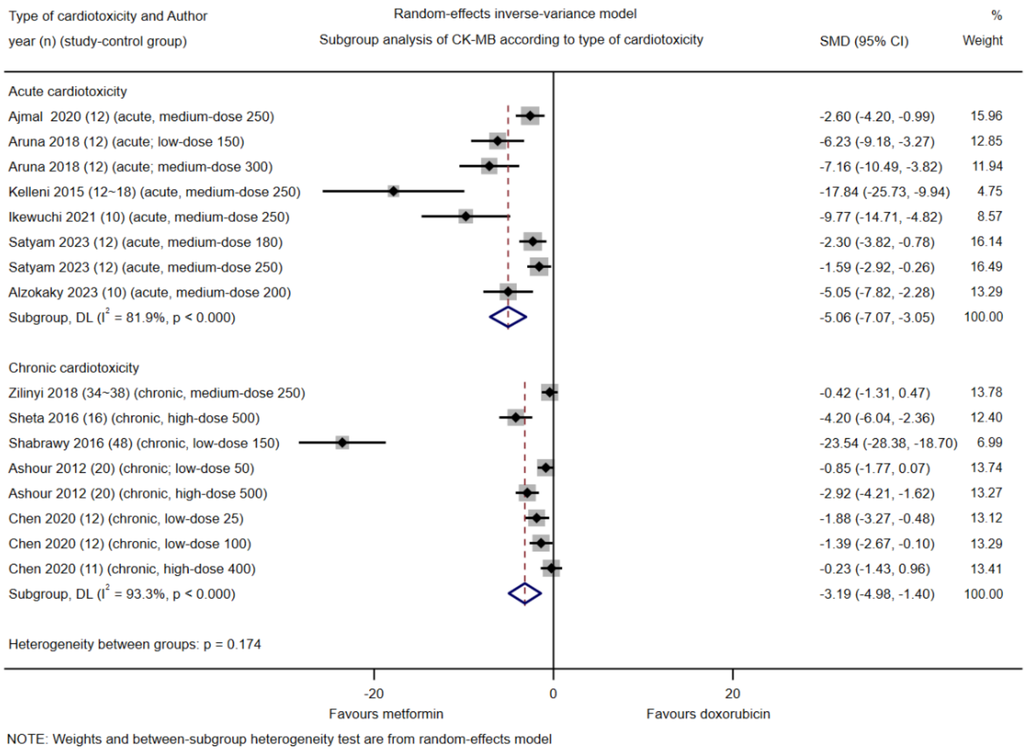

Fig. S6 Subgroup analysis according to type of cardiotoxicity for serum CK-MB

Note: Subgroup analysis discovered that type of cardiotoxicity did not contribute to the heterogeneity for serum CK-MB.

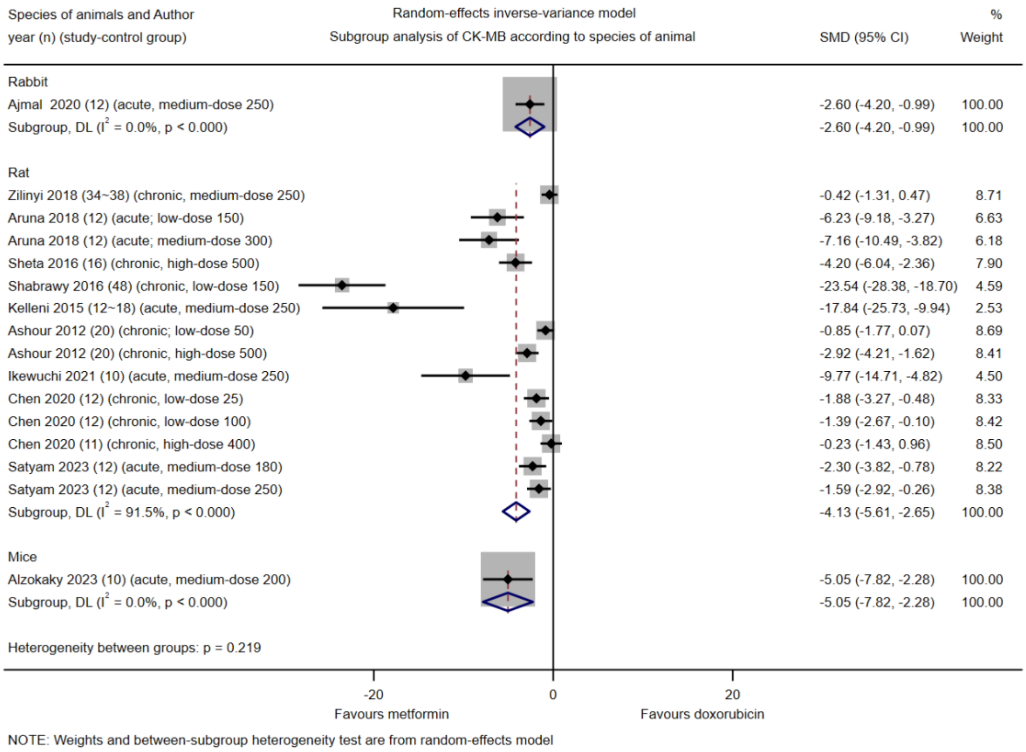

Fig. S7 Subgroup analysis according to species of animal for serum CK-MB

Note: Subgroup analysis discovered that species of animal did not contribute to the heterogeneity for serum CK-MB.

Fig. S8 Subgroup analysis according to study designs for serum CK-MB

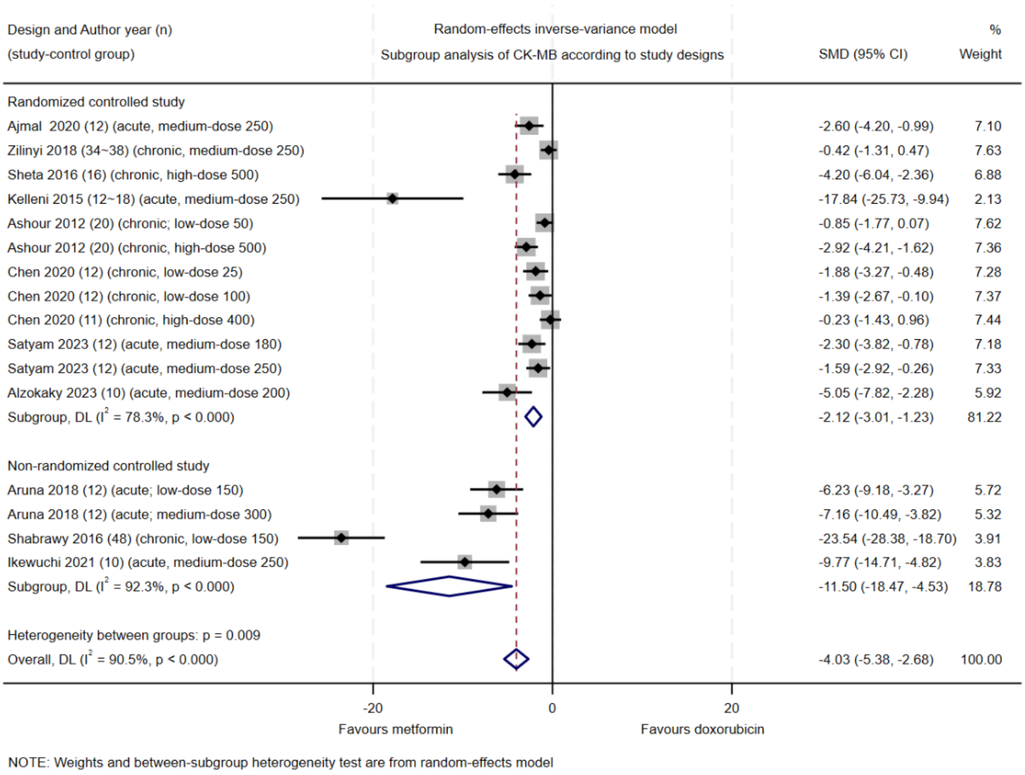

Fig. S8 Subgroup analysis according to study designs for serum CK-MB

Note: Subgroup analysis discovered that study designs did not contribute to the heterogeneity for serum CK-MB.

8.2 Meta-regression analysis for serum creatine CK-MB

Meta-regression	Number of obs = 16
REML estimate of between-study variance	tau2 = 28.32
% residual variation due to heterogeneity	I-squared_res = 88.32%
Proportion of between-study variance explained	Adj R-squared = 10.83%
Joint test for all covariates	Model F(4,11) = 1.20
With Knapp-Hartung modification	Prob > F = 0.3660

_ES	Coefficient	Std. err.	t	P>|t|	[95% conf.	interval]
doselevelofmet	-.042721	2.125629	-0.02	0.984	-4.721198	4.635756
kindofcardiotoxicity	-.8745239	3.371677	-0.26	0.800	-8.295535	6.546487
kindofanimal	.3783372	3.123635	0.12	0.906	-6.496738	7.253412
studydesigns	8.180466	4.270374	1.92	0.082	-1.218563	17.5795
_cons	-10.75052	6.994365	-1.54	0.153	-26.14501	4.643977

Note: meta-regression analysis failed to reveal the source of heterogeneity of CK-MB.

9. Subgroup- and meta-regression analysis for serum cardiac troponin I (cTnI)

9.1 Subgroup analysis for serum cTnI

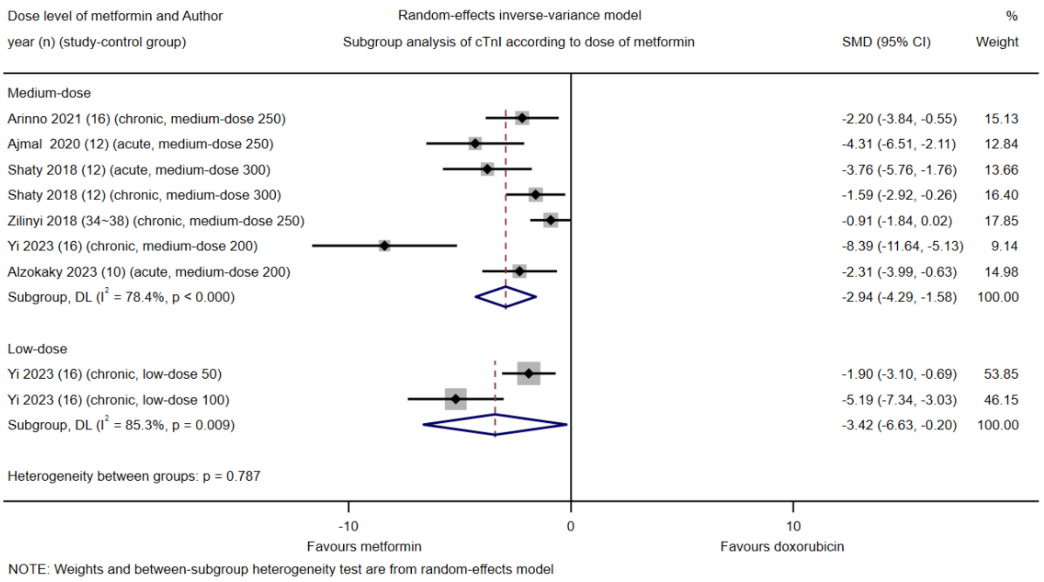

Fig. S9 Subgroup analysis according to dose of metformin for serum cTnI

Note: Subgroup analysis discovered that dose of metformin did not contribute to the heterogeneity for serum cTnI.

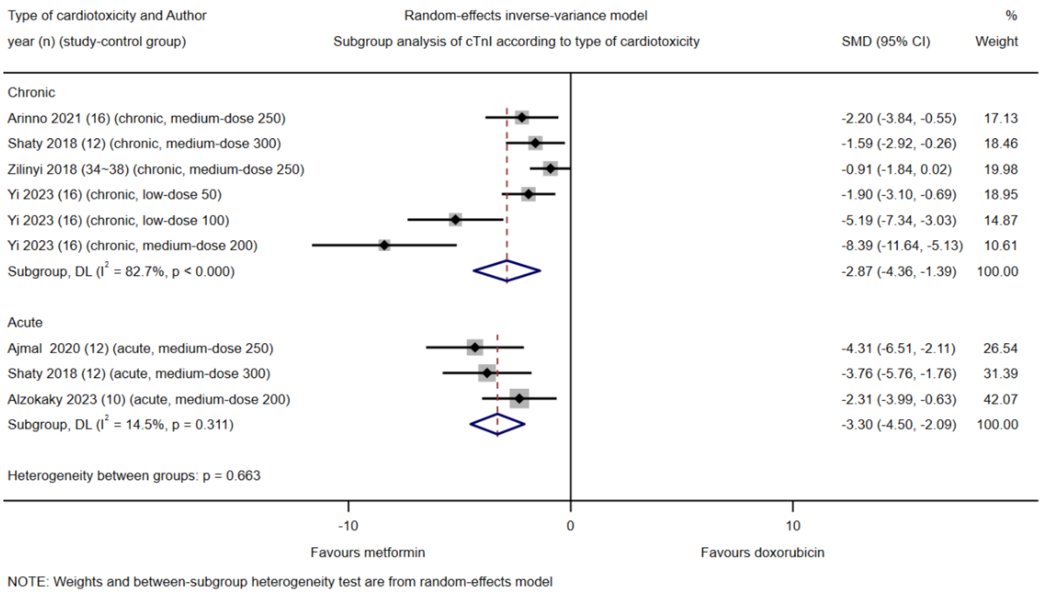

Fig. S10 Subgroup analysis according to type of cardiotoxicity for serum cTnI

Note: Subgroup analysis discovered that type of cardiotoxicity did not contribute to the heterogeneity for serum cTnI.

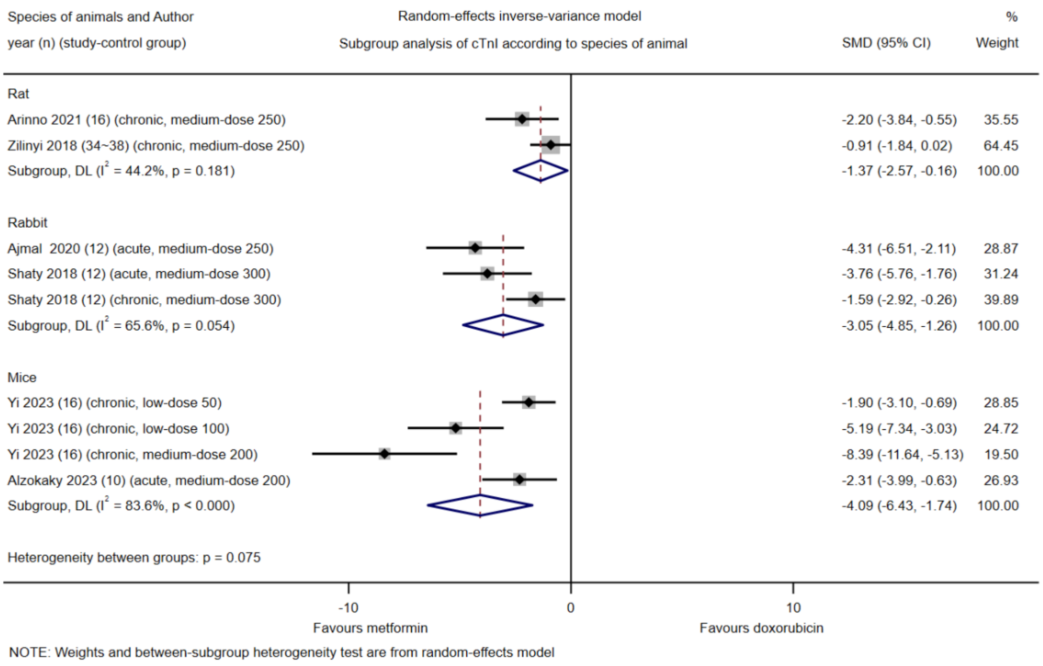

Fig. S11 Subgroup analysis according to species of animal for serum cTnI

Note: Subgroup analysis discovered that species of animal did not contribute to the heterogeneity for serum cTnI.

9.2 Meta-regression analysis for serum cTnI

REML estimate of between-study variance	tau2	=	5.54
% residual variation due to heterogeneity	I-squared_res	=	81.24%
Proportion of between-study variance explained	Adj R-squared	=	-83.79%
Joint test for all covariates	Model F(3,5)	=	0.12
With Knapp-Hartung modification	Prob > F	=	0.9418

_ES	Coefficient	Std. err.	t	P>|t|	[95% conf.	interval]
doselevelofmet	.360493	2.331795	0.15	0.883	-5.633577	6.354563
kindofcardioto~y	.1228564	2.428884	0.05	0.962	-6.120789	6.366502
kindofanimal	-.7397505	1.409287	-0.52	0.622	-4.362437	2.882936
_cons	-2.437769	4.477415	-0.54	0.610	-13.94733	9.071793

Note: meta-regression analysis failed to reveal the source of heterogeneity of cTnI**.**

As all studies included in the data synthesis of cTnI were randomized controlled trials, subgroup analysis and meta-regression analysis were not conducted based on the study design.

10. Sensitivity analysis of primary efficacy outcomes

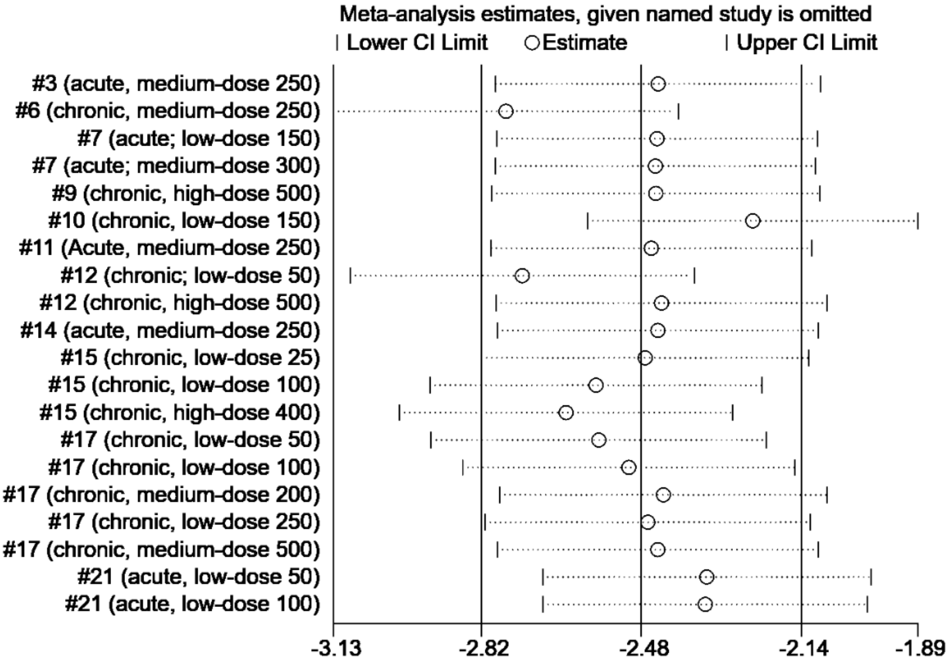

Fig. S12 Sensitivity analysis using "leave-one-out" method for serum LDH

Note: Sensitivity analysis using the leave-one-out method indicated that the pooled result of serum LDH was stable.

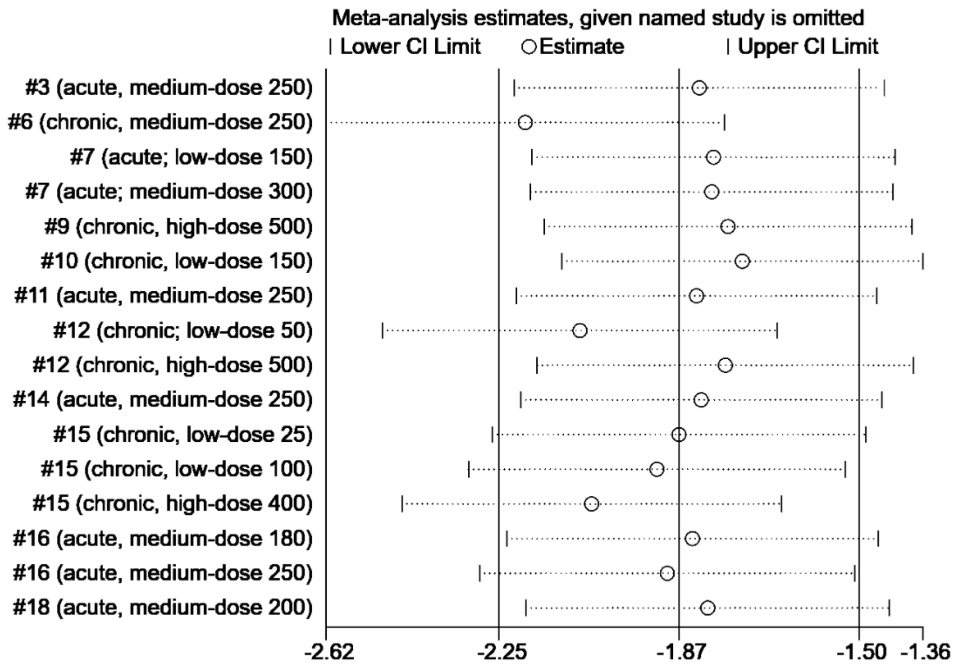

Fig. S13 Sensitivity analysis using "leave-one-out" method for serum CK-MB

Note: Sensitivity analysis using the leave-one-out method indicated that the pooled result of serum CK-MB was stable.

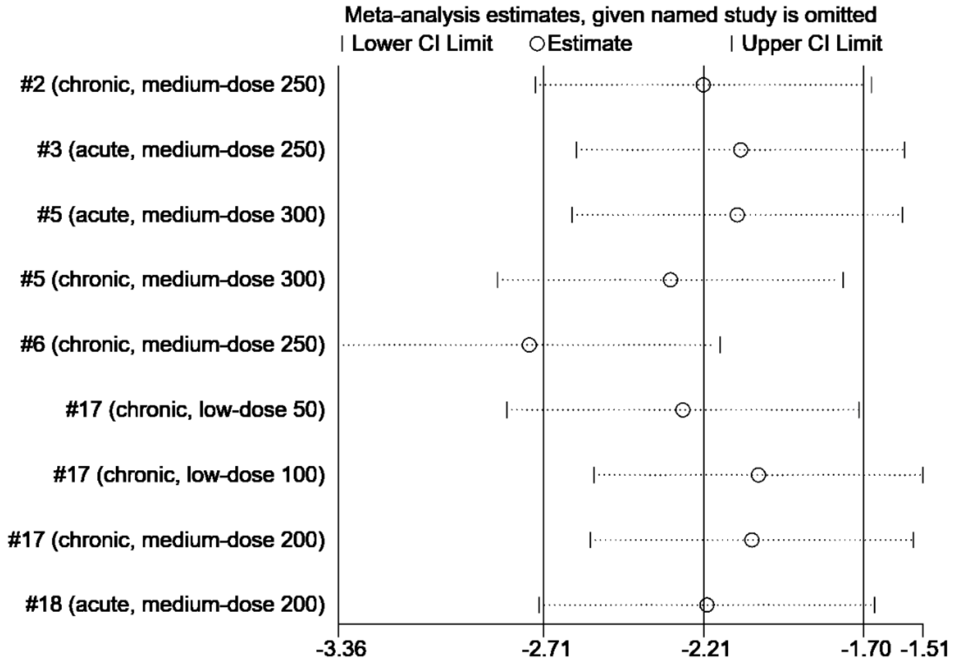

Fig. S14 Sensitivity analysis using "leave-one-out" method for serum cTnI

Note: Sensitivity analysis using the leave-one-out method indicated that the pooled result of serum cTnI was stable.

11. Data synthesis and heterogeneity analysis of secondary outcomes

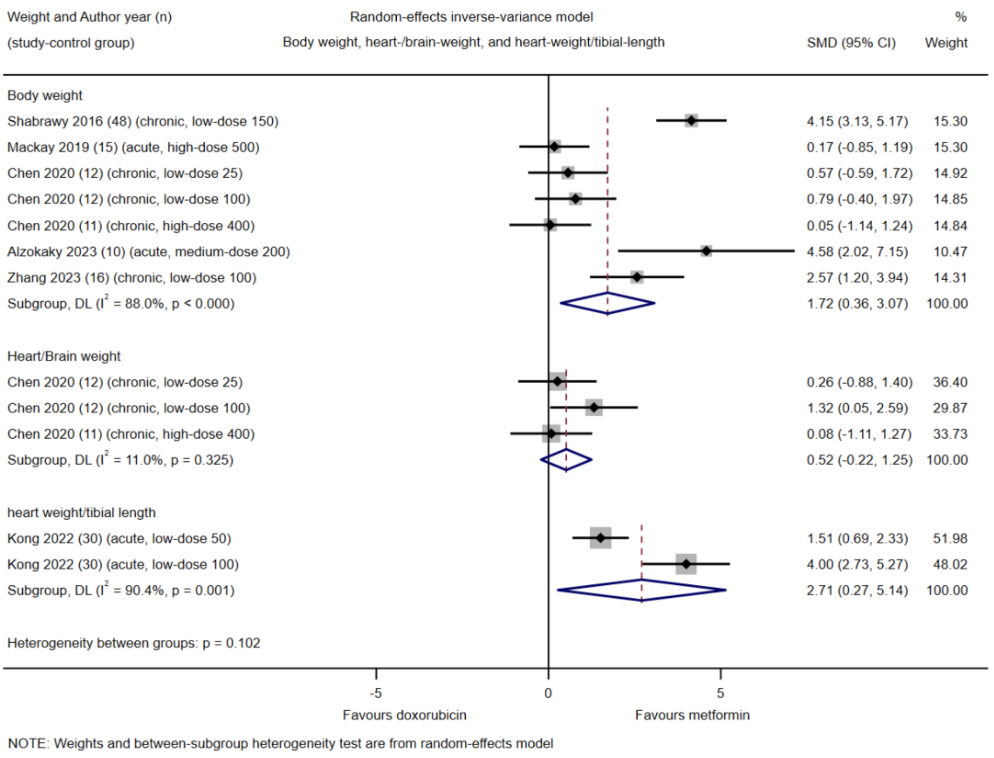

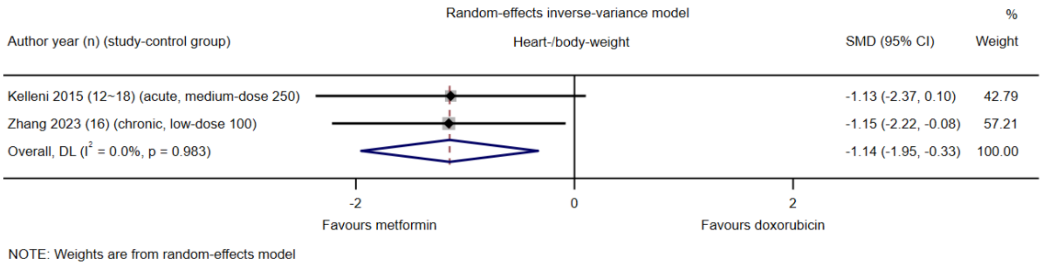

Fig. S15 Forest plot of body weight and relative value of heart weight

Note: Met improves heart weights significantly larger than tibial length, smaller than the body weight, and equal to the brain weight.

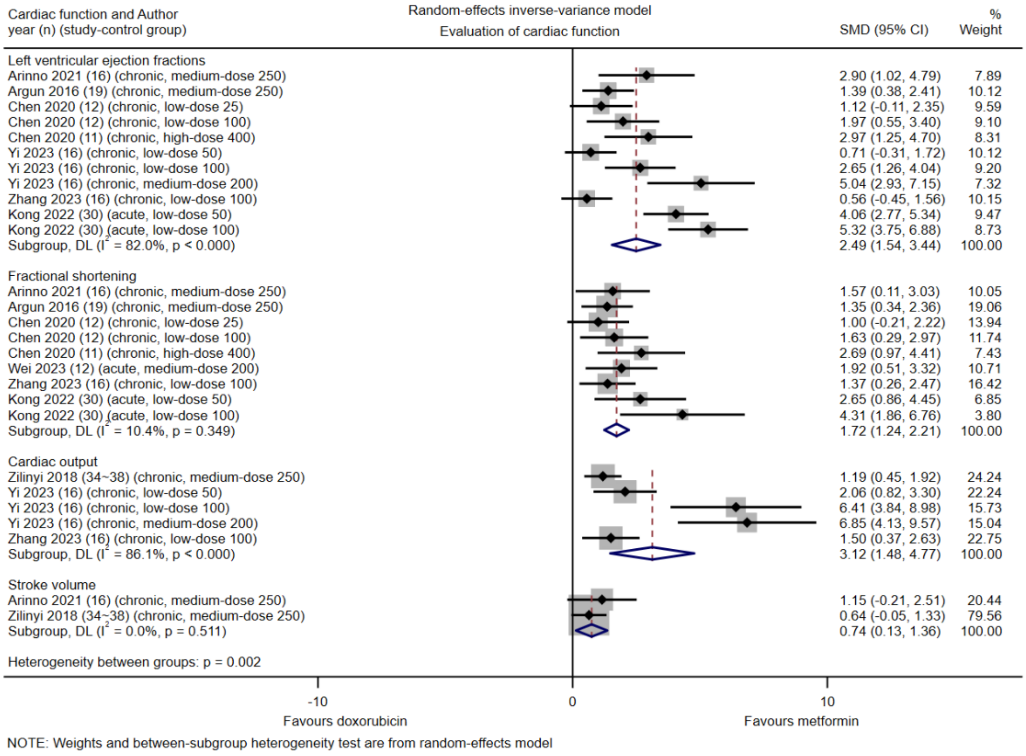

Fig. S16 Forest plot of cardiac function

Note: Metformin improves cardiac function in animals with Dox-induced cardiotoxicity.

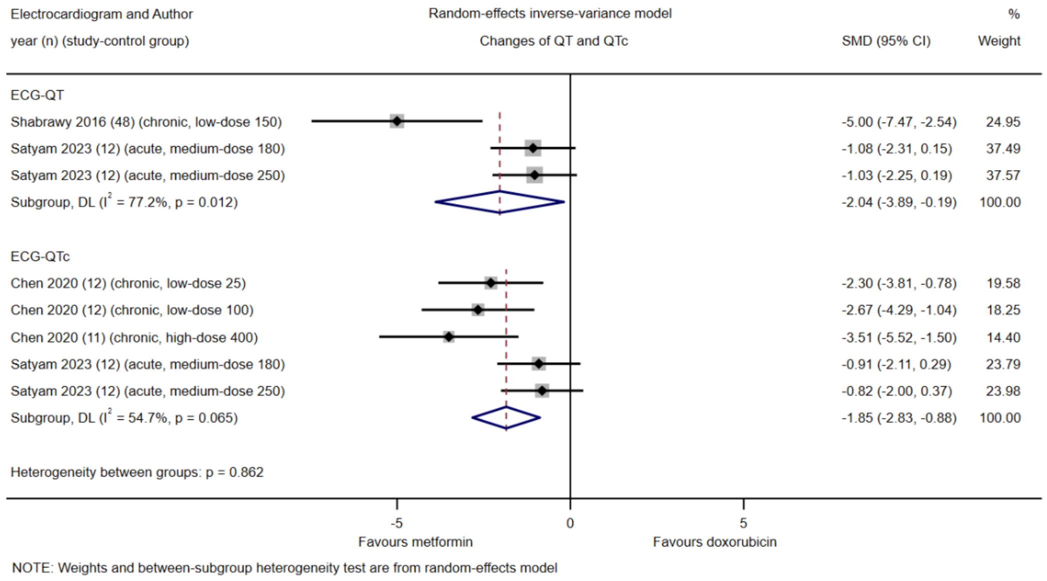

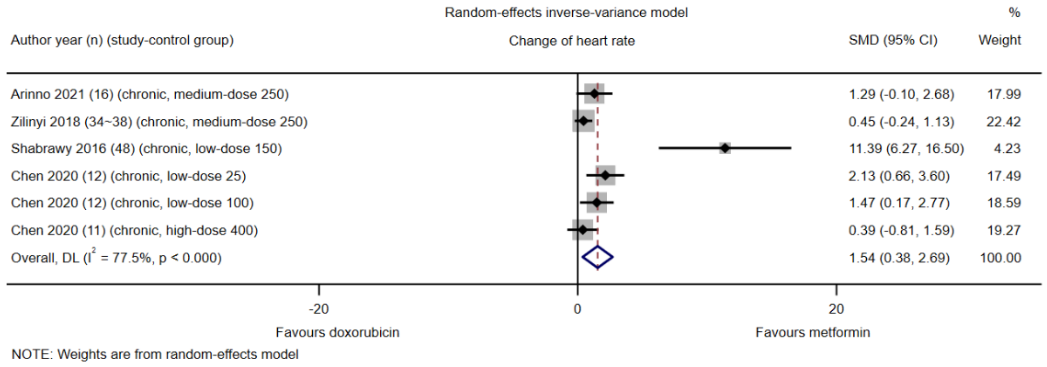

Fig. S17 Forest plot of characteristics of electrocardiogram (above) and heart rates (below)

Note: Met treated animals exhibited significant reduction in QT interval, QTc interval, and improvement in heart rate compared to the animals with Dox-induced cardiotoxicity.

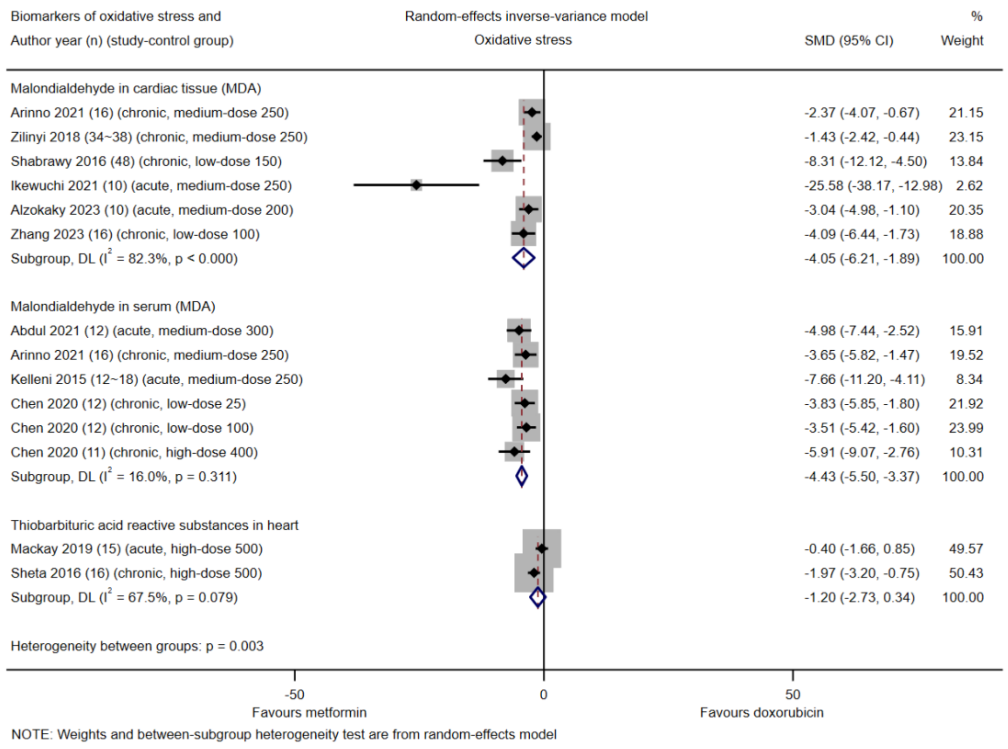
Biomarkers of oxidative stress and
Author year (n) (study-control group)
Random-effects inverse-variance model
Oxidative stress
SMD (95% CI)
%
Weight
Malondialdehyde in cardiac tissue (MDA)
Arinno 2021 (16) (chronic, medium-dose 250) -2.37 (-4.07, -0.67) 21.15
Zilinyi 2018 (34~38) (chronic, medium-dose 250) -1.43 (-2.42, -0.44) 23.15
Shabrawy 2016 (48) (chronic, low-dose 150) -8.31 (-12.12, -4.50) 13.84
Ikewuchi 2021 (10) (acute, medium-dose 250) -25.58 (-38.17, -12.98) 2.62
Alzokaky 2023 (10) (acute, medium-dose 200) -3.04 (-4.98, -1.10) 20.35
Zhang 2023 (16) (chronic, low-dose 100) -4.09 (-6.44, -1.73) 18.88
Subgroup, DL (I^2 = 82.3%, p < 0.000) -4.05 (-6.21, -1.89) 100.00
Malondialdehyde in serum (MDA)
Abdul 2021 (12) (acute, medium-dose 300) -4.98 (-7.44, -2.52) 15.91
Arinno 2021 (16) (chronic, medium-dose 250) -3.65 (-5.82, -1.47) 19.52
Kelleni 2015 (12~18) (acute, medium-dose 250) -7.66 (-11.20, -4.11) 8.34
Chen 2020 (12) (chronic, low-dose 25) -3.83 (-5.85, -1.80) 21.92
Chen 2020 (12) (chronic, low-dose 100) -3.51 (-5.42, -1.60) 23.99
Chen 2020 (11) (chronic, high-dose 400) -5.91 (-9.07, -2.76) 10.31
Subgroup, DL (I^2 = 16.0%, p = 0.311) -4.43 (-5.50, -3.37) 100.00
Thiobarbituric acid reactive substances in heart
Mackay 2019 (15) (acute, high-dose 500) -0.40 (-1.66, 0.85) 49.57
Sheta 2016 (16) (chronic, high-dose 500) -1.97 (-3.20, -0.75) 50.43
Subgroup, DL (I^2 = 67.5%, p = 0.079) -1.20 (-2.73, 0.34) 100.00
Heterogeneity between groups: p = 0.003
-50
0
50
Favours metformin
Favours doxorubicin
NOTE: Weights and between-subgroup heterogeneity test are from random-effects model

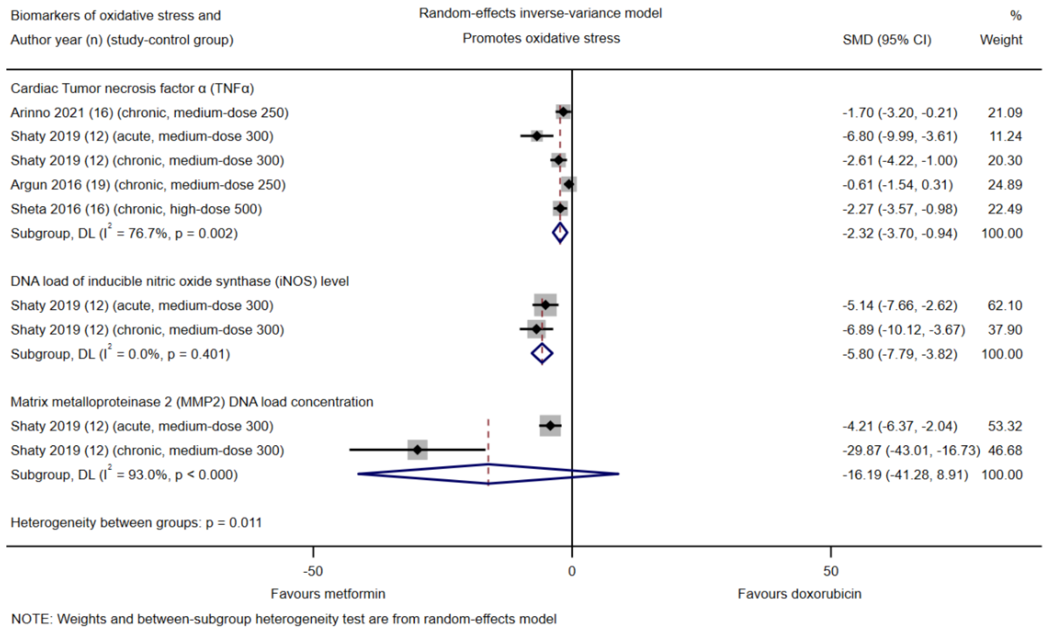
Biomarkers of oxidative stress and
Author year (n) (study-control group)
Random-effects inverse-variance model
Promotes oxidative stress
SMD (95% CI)
%
Weight
Cardiac Tumor necrosis factor α (TNFα)
Arinno 2021 (16) (chronic, medium-dose 250) -1.70 (-3.20, -0.21) 21.09
Shaty 2019 (12) (acute, medium-dose 300) -6.80 (-9.99, -3.61) 11.24
Shaty 2019 (12) (chronic, medium-dose 300) -2.61 (-4.22, -1.00) 20.30
Argun 2016 (19) (chronic, medium-dose 250) -0.61 (-1.54, 0.31) 24.89
Sheta 2016 (16) (chronic, high-dose 500) -2.27 (-3.57, -0.98) 22.49
Subgroup, DL (I^2 = 76.7%, p = 0.002) -2.32 (-3.70, -0.94) 100.00
DNA load of inducible nitric oxide synthase (iNOS) level
Shaty 2019 (12) (acute, medium-dose 300) -5.14 (-7.66, -2.62) 62.10
Shaty 2019 (12) (chronic, medium-dose 300) -6.89 (-10.12, -3.67) 37.90
Subgroup, DL (I^2 = 0.0%, p = 0.401) -5.80 (-7.79, -3.82) 100.00
Matrix metalloproteinase 2 (MMP2) DNA load concentration
Shaty 2019 (12) (acute, medium-dose 300) -4.21 (-6.37, -2.04) 53.32
Shaty 2019 (12) (chronic, medium-dose 300) -29.87 (-43.01, -16.73) 46.68
Subgroup, DL (I^2 = 93.0%, p < 0.000) -16.19 (-41.28, 8.91) 100.00
Heterogeneity between groups: p = 0.011
-50
0
50
Favours metformin
Favours doxorubicin
NOTE: Weights and between-subgroup heterogeneity test are from random-effects model

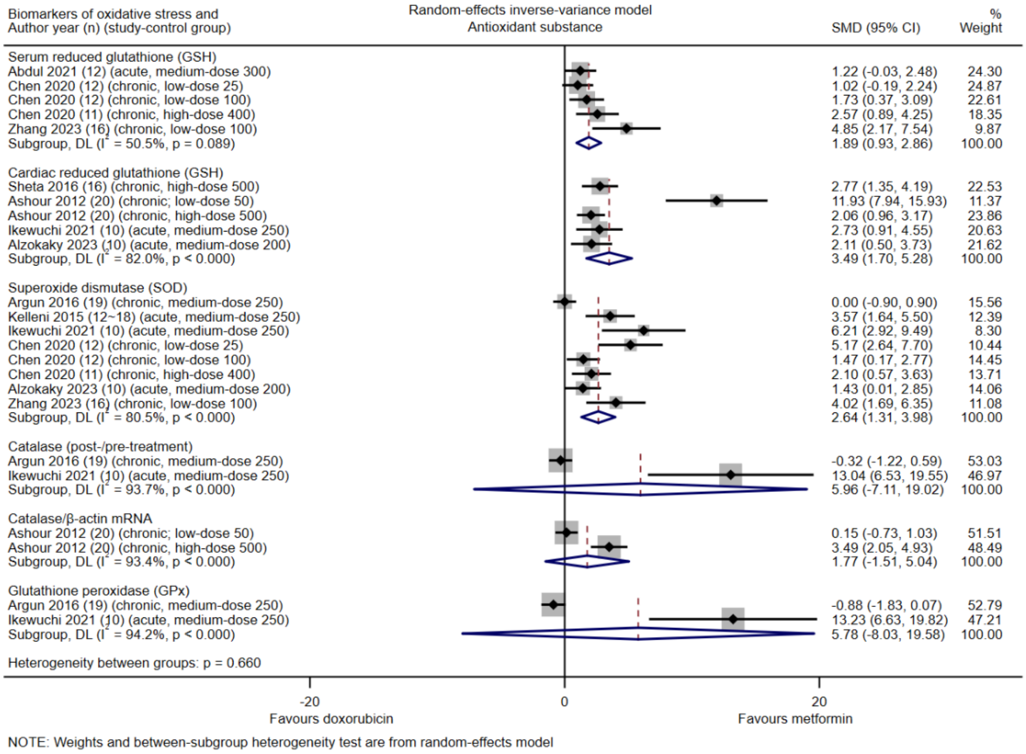

Fig. S18 Forest plot of oxidative stress hypothesis (A, B, C)

Note: Metformin reduces oxidative stress, reduces substances that promote oxidative stress, increases reducing substances, or has a tendency to do so.

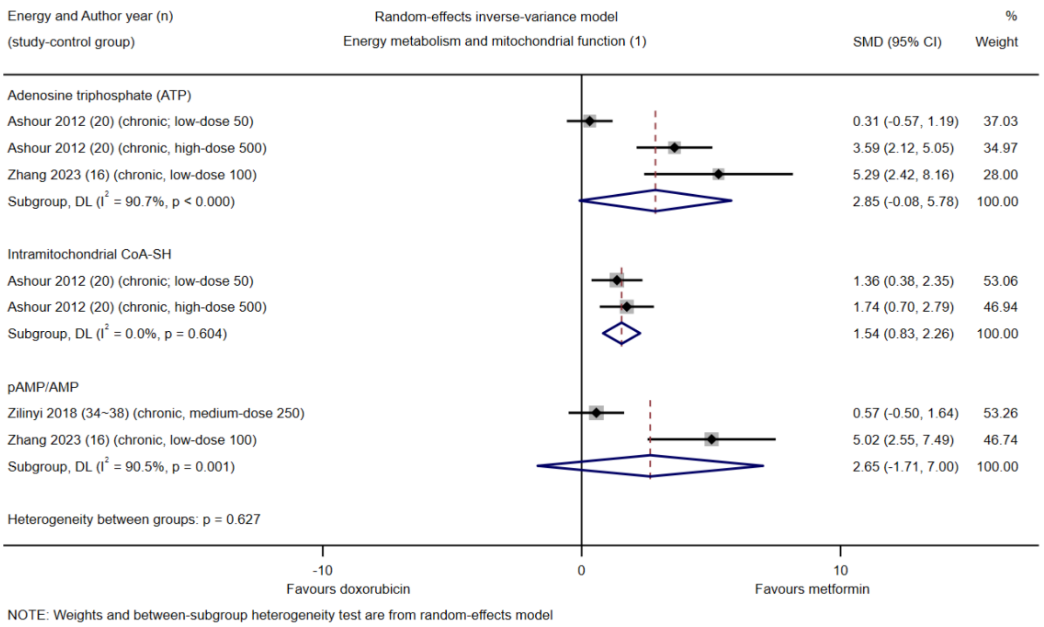

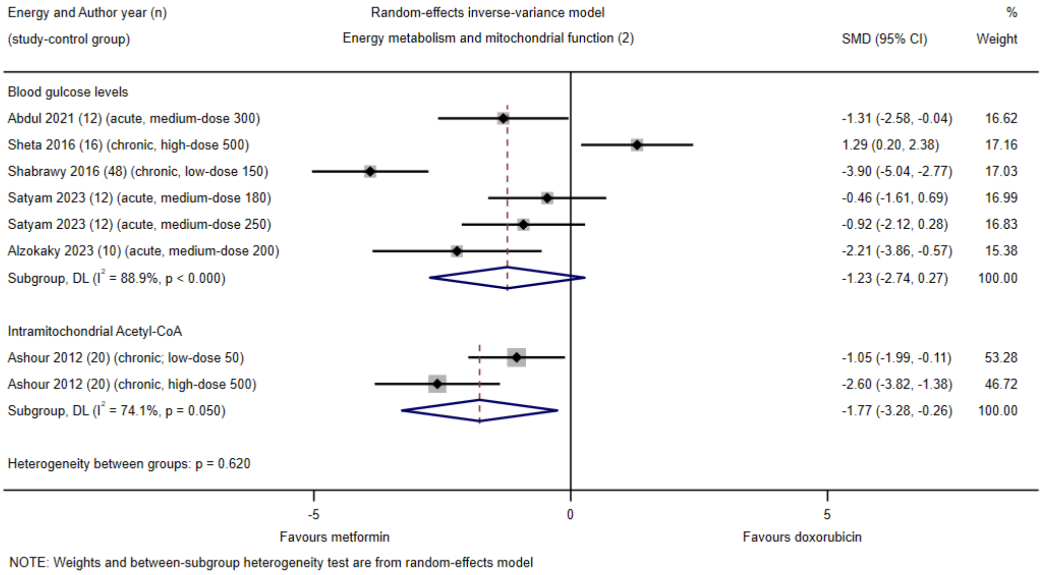

Fig. S19 Forest plot of alleviating energy starvation and preserving mitochondrial function hypothesis (A, B)

Note: Metformin alleviates energy starvation and preserves mitochondrial function in animals with Dox-induced cardiotoxicity, or has a tendency to do so.

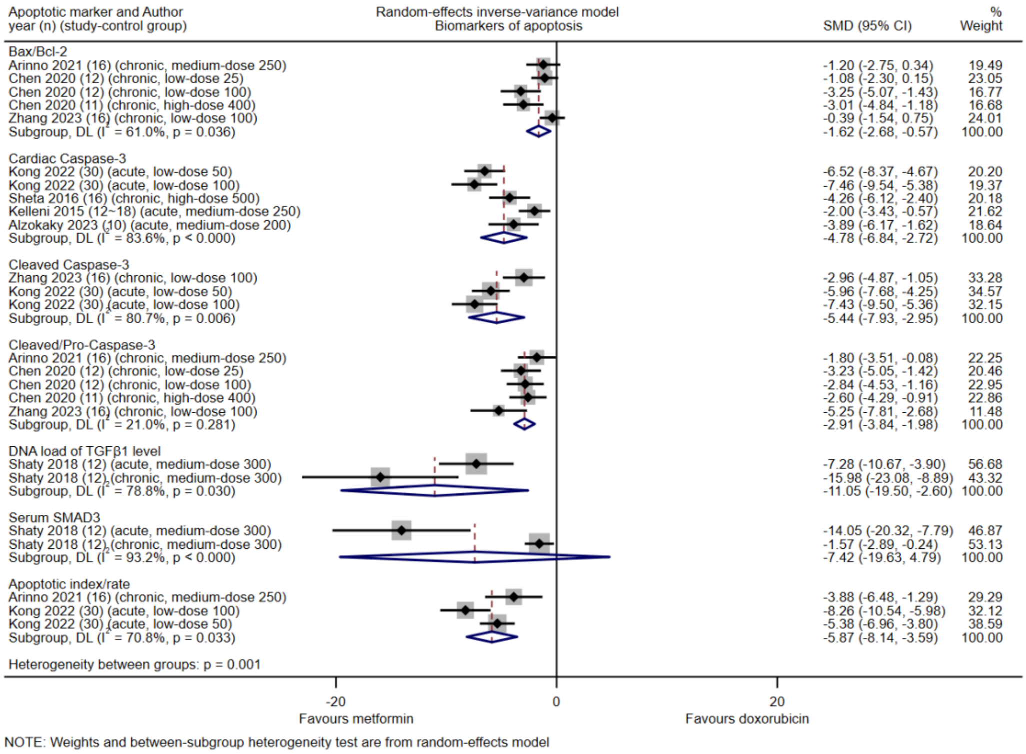

Fig. S20 Forest plot of apoptosis hypothesis

Note: Metformin reduced apoptotic markers in animals with Dox-induced cardiotoxicity.

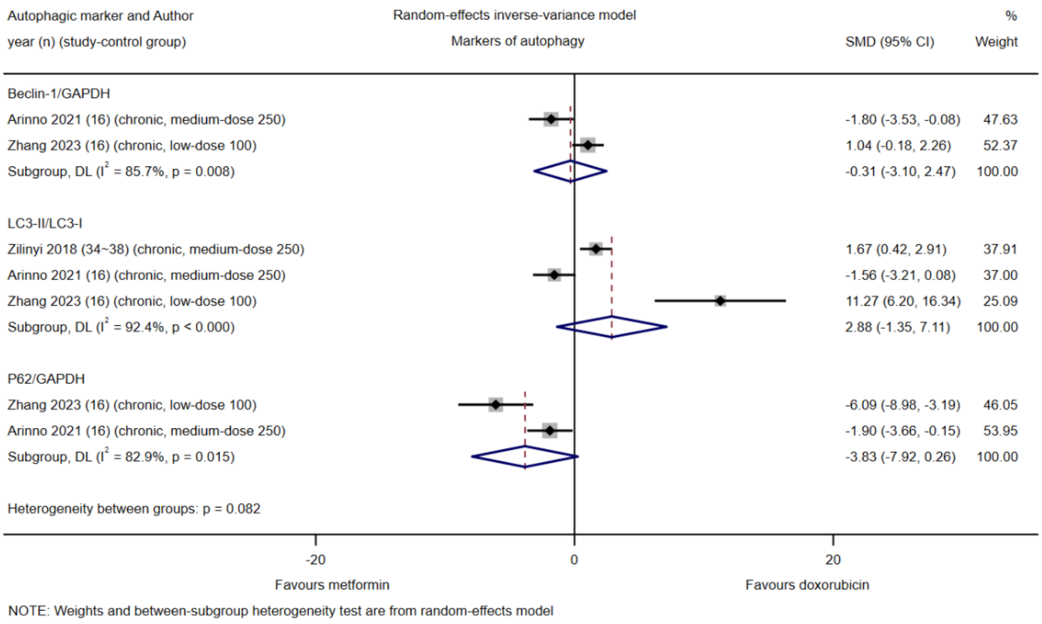

Fig. S21 Forest plot of autophagy hypothesis

Note: Metformin showed a tendency to improve autophagy markers in animals with Dox-induced cardiotoxicity.

12. Funnel plot for publication bias of the primary efficacy outcomes

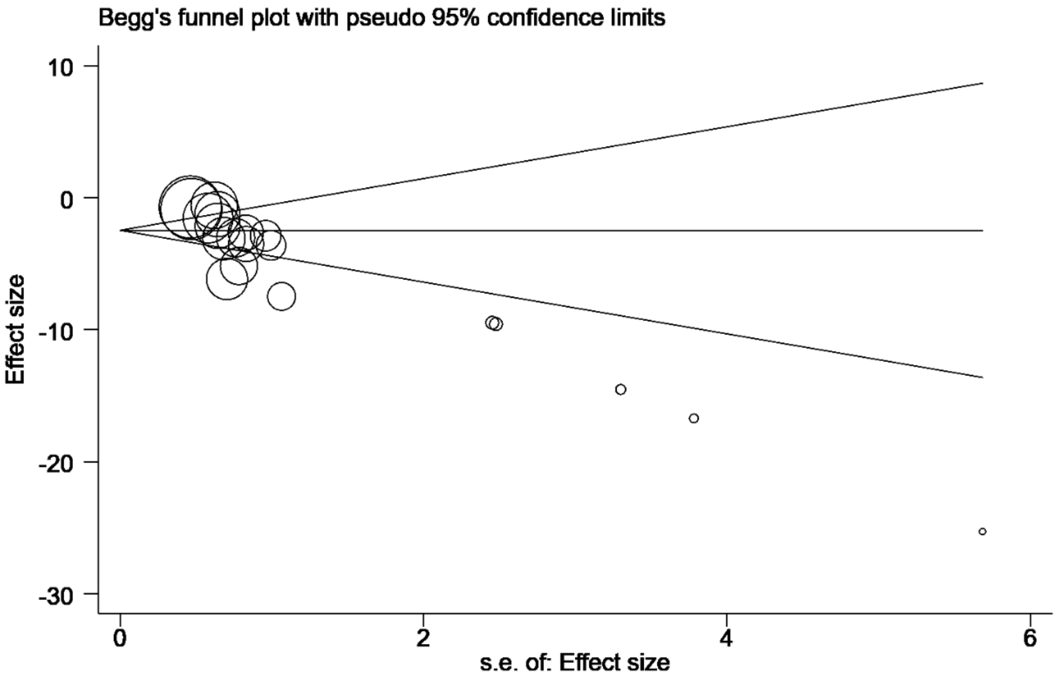

Fig. S22 serum lactate dehydrogenase (LDH)

Note: The funnel plot of serum LDH is obviously asymmetrical, indicating the existence of significant publication bias.

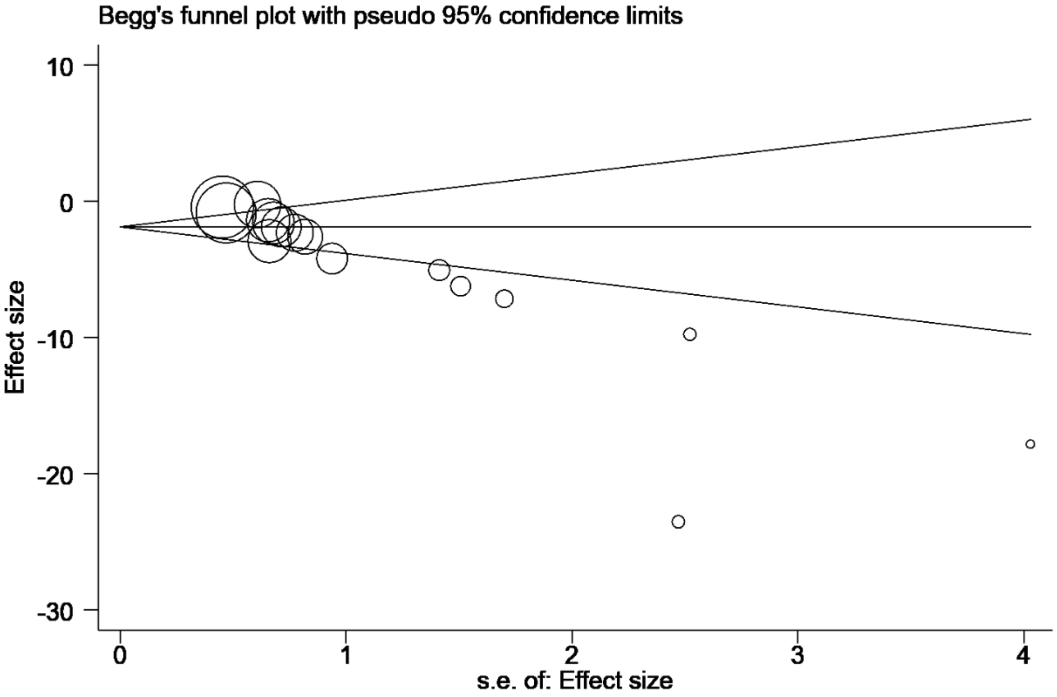

Fig. S23 serum creatine kinase-myocardial band (CK-MB)

Note: The funnel plot of serum CK-MB is obviously asymmetrical, indicating the existence of significant publication bias.

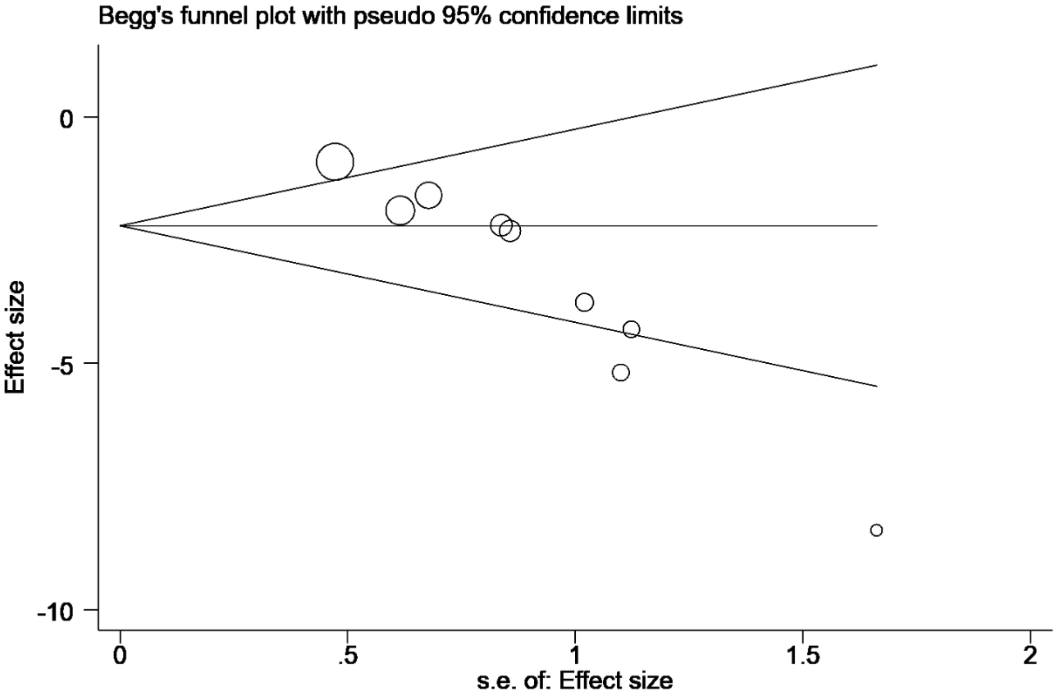

Fig. S24 The funnel plot of serum cardiac troponin I (cTnI)

Note: The funnel plot of serum cTnI is obviously asymmetrical, indicating the existence of significant publication bias.
